# Age-related changes in upper limb motion during typical development

**DOI:** 10.1371/journal.pone.0198524

**Published:** 2018-06-06

**Authors:** Cristina Simon-Martinez, Gabriela Lopes dos Santos, Ellen Jaspers, Ruth Vanderschueren, Lisa Mailleux, Katrijn Klingels, Els Ortibus, Kaat Desloovere, Hilde Feys

**Affiliations:** 1 KU Leuven - University of Leuven, Department of Rehabilitation Sciences, Leuven, Belgium; 2 Laboratory of Neurological Physiotherapy Research, Department of Physiotherapy, Federal University of São Carlos (UFSCar), São Carlos, São Paulo, Brazil; 3 Neural Control of Movement Lab, Department of Health Sciences and Technology, ETH Zurich, Switzerland; 4 Rehabilitation Research Centre, BIOMED, Hasselt University, Diepenbeek, Belgium; 5 KU Leuven - University of Leuven, Department of Development and Regeneration, Leuven, Belgium; 6 Clinical Motion Analysis Laboratory, University Hospital Leuven, Pellenberg, Belgium; Nanyang Technological University, SINGAPORE

## Abstract

**Background and aim:**

Understanding the maturation of upper limb (UL) movement characteristics in typically developing (TD) children is key to explore UL deficits in those with neurodevelopmental disorders. Three-dimensional motion analysis (3DMA) offers a reliable tool to comprehensively evaluate UL motion. However, studies thus far mainly focused on specific pre-defined parameters extracted from kinematic waveforms. Here, we investigated age-related differences in UL movement characteristics over the entire movement cycle in TD children.

**Participants and methods:**

We assessed the non-dominant UL of 60 TD children (mean age 10y3m±3y1m) using 3DMA during eight tasks: reaching (forwards (RF), upwards (RU), sideways (RS)), reach-to-grasp (sphere (RGS), vertical cylinder (RGV)) and activities-of-daily-living mimicking tasks (hand-to-head (HTH), hand-to-mouth (HTM), hand-to-shoulder (HTS)). We investigated differences between four age-groups (5-7y, 8-10y, 11-12y, 13-15y) in: (1) spatiotemporal parameters (movement duration, peak velocity, time-to-peak velocity and trajectory straightness), and (2) 12 UL joint angles, using Statistical Parametric Mapping (SPM).

**Results:**

We found that the 5-7y children moved with lower peak velocity and less straight trajectories compared to the 11-12y group (peak velocity: RS, HTS, p<0.01; trajectory: RU, RS, RGV, HTS, p<0.01) and the 13-15y group (peak velocity: RF, RS, RGS, RGV, HTH, HTS, p<0.01; trajectory, all tasks, p<0.01). The 5-7y children showed increased scapular protraction compared to older children (8-10y and 11-12y, HTS), as well as increased scapular medial rotation compared to the 13-15y group (RGS). During RU, the 5-7y children moved more towards the frontal plane (shoulder), unlike the 13-15y group. Lastly, the 5-7y group used less elbow flexion than older children (11-12y and 13-15y) during HTH and HTS.

**Discussion and conclusion:**

In conclusion, our results point toward a maturation in UL movement characteristics up to age 11-12y, when UL motion seemed to reach a plateau. The reference values provided in this study will help to further optimize the interpretation of UL deficits in children with neurodevelopmental disorders.

## Introduction

Upper limb (UL) motion is essential for the motor, social and cognitive development of children as it allows interaction between the individual and his/her environment. The development of UL motion may be compromised by the presence of a neurodevelopmental disorder, such as cerebral palsy, Duchenne’s muscular dystrophy or brachial plexus palsy, which may negatively influence the performance of activities of daily living (ADL) and potentially affect the child’s participation and quality of life [1,2].

In typically developing (TD) children, the development of reaching and grasping skills has been investigated using three-dimensional motion analysis (3DMA). Previous studies have shown a considerable improvement of smoothness and trajectory straightness throughout the first years of life [3–6] and reported a mature coordination between arm and trunk movements around the age of 4 years [3]. However, further development continues until the age of 8–10 years, whereby children show smoother and more stable hand trajectories [3,7]. UL movement maturation reaches a plateau around the age of 11 years [3,4,6,8]. The vast amount of 3DMA literature on the development of reaching tasks is contrasted by only a few studies that reported age-differences in UL kinematics during ADL-mimicking tasks [9,10].

Furthermore, the normative values reported by previous studies focused on extracting specific parameters from the kinematic waveform (e.g. active range of motion and endpoint angles). Although these parameters have commonly been used to investigate UL motion in children and adults [11–14], they allow only an incomplete interpretation as information about the entire UL movement pattern is omitted. Moreover, extracting only those data points where the differences are maximum could lead to false-positive results and therefore to a biased interpretation of the norm values [15]. An alternative analysis approach designed for continuous field analysis has recently been implemented in biomechanics, i.e. Statistical Parametric Mapping (SPM) [16]. SPM allows investigating the entire kinematic waveform as it takes the interdependency of data points into account by using random field theory. Therefore, this analysis approach reduces the risk of incorrectly rejecting the null hypothesis. However, the potential value of SPM in investigating age-related differences in UL motion has not been explored yet.

Here, we explored age-related differences in UL kinematics in a large cohort of TD children. We hypothesized that spatiotemporal parameters would change during reaching, reach-to-grasp and ADL-mimicking tasks, resulting in reduced total movement duration and time to peak velocity, and increased peak velocity and trajectory straightness, in growing TD children. Moreover, we hypothesized that additional kinematic changes related to age would be observed by using SPM analyses. Specifically, and supported by data from the literature [3,8,9], we hypothesized that a maturation plateau would be reached around the age of 11 years old.

## Materials and methods

### Participants

An observational, prospective, cohort study was set up for which TD children aged 5–15 years were recruited via schools, youth movements and colleagues in Flanders (Belgium) between 2010 and 2016. Children should have normal or corrected-to-normal vision during the task performance. Furthermore, oral interview with the parents confirmed that the children had a typical development. Children with a history of neurological or musculoskeletal disorders at any time point were excluded. Recruitment via sport clubs or music schools was avoided to warrant a representative sample for the normal population and exclude potential impact of highly skilled UL activities. Children who were willing to participate and fulfilled the inclusion criteria were prospectively enrolled in the study, by attempting to keep and equal number of participants and a sex balance within each age group. This resulted in 60 TD children (mean age (SD) 10y3m (3y1m), 36 boys, 53 right-handed) who underwent a 3DMA. Prior to participation, children gave their verbal assent to partake and parents signed the informed consent, in accordance with the Declaration of Helsinki. This study was approved by the Medical Ethics Committee UZ KU Leuven (S50480 and S55555). All measurements were conducted by two experienced physiotherapists (CSM and EJ) at the Clinical Motion Analysis Laboratory of the University Hospitals Leuven (campus Pellenberg, Belgium).

### Experimental procedures

First, UL dominance was determined by asking the children to draw (in young children, 5-7y) or to write (in older children, >7y). Similarly to other studies, the non-dominant UL was measured, to serve as a reference for the impaired side of children with UL deficits [17–20]. Previous studies have shown that TD children move with shorter movement times [21] and different kinematic joint angles at point of task achievement [9] with the dominant compared to the non-dominant UL. To ameliorate these differences, we chose to measure the non-dominant UL in this study.

3DMA was conducted while seated, using a custom-made chair to ensure 90° of hip and knee flexion through an adjustable lower back and feet support. Next, 17 markers were mounted on the trunk, acromion, upper and lower arm, and hand; and anatomical landmarks of interest were recording during several static trials, following the ISB-recommendations and the protocol by Jaspers et al. [22,23]. Motion was recorded using 12 or 15 Vicon infrared cameras (Oxford Metrics, Oxford, UK), sampling at 100 Hz. Children were then instructed to perform a movement protocol consisting of eight tasks: three reaching (forwards (RF), upwards (RU) and sideways (RS)), two reach-to-grasp (a sphere (RGS) and a vertically oriented cylinder (RGV)), and three ADL-mimicking tasks (hand-to-head (HTH, grooming), hand-to-mouth (HTM, eating) and hand-to-shoulder (HTS, dressing)). Reaching and reach-to-grasp tasks were performed at shoulder height, except RU, which was executed at eye-height; reaching distance was based on the arm length (see also [23]). Each task was recorded twice, and during each task recording children were instructed to repeat the movement four times, resulting in a total of eight movement repetitions per task. All tasks started with the non-dominant hand on the ipsilateral knee and were performed at self-selected speed, by instructing the participants to “perform the task at your own pace”, as this pace most likely represents their typical behavior during daily life activities and it is commonly used in literature [24]. Performing the tasks at self-selected speed allows us to investigate changes in speed with age. Moreover, as speed plays a major role in motor control in children with disabilities, potentially compromising accuracy and the movement pattern itself, performing the tasks at self-selected speed in this study will permit a later comparison with children with disabilities. This protocol was proven reliable in TD children [23].

### Data processing

To avoid variability due to start and stop strategies, the first and last repetition of each recording were excluded from further analysis, resulting in four cycles per task. 3D marker coordinates were processed using Vicon Nexus 1.8.5 software (Oxford Metrics, Oxford, UK). After data filtering (Woltring routine with a predicted mean squared error of 10 mm^2^ [25]), start and endpoint of each movement cycle were identified. The start point was always the position with the hand on the ipsilateral knee, whereas the endpoint varied depending on the task: touching the object with the palm of the hand (RF, RU, and RS); grasping the object (RGS and RGV); touching the top of the head (HTH), the mouth (HTM) or the contralateral shoulder (HTS). Movement cycles were time normalized (0–100%) and the UL spatiotemporal and kinematic calculations were computed with U.L.E.M.A. software (version 1.1.9, Matlab-based open source software, available for download at https://github.com/u0078867/ulema-ul-analyzer) [18]. Based on time events of the third metacarpal (anatomical landmark), the following spatiotemporal parameters were extracted: total movement duration (seconds), time to peak velocity (%), peak velocity (m/s), and trajectory straightness (non-dimensional). This parameter equals the ratio between the length of the hand marker path and the straight line connecting the first and last marker position. Kinematic data of five joints (12 joint angles) was also calculated: trunk (flexion-extension, lateral flexion and axial rotation), scapula (anterior-posterior tilting, pro-retraction and medial-lateral rotation), shoulder (elevation plane, elevation and rotation), elbow (flexion-extension and pro-supination) and wrist (flexion-extension). The shoulder rotation center was estimated using a linear regression equation [26]. After UL kinematic calculations, we compared, per task, the kinematic data of each cycle to the mean of the three remaining cycles by computing the root mean square error (RMSE) over the entire movement cycle and averaged it across data points. To maximize performance repeatability, only the three cycles with lowest RMSE were retained for further statistical analysis (routine implemented in U.L.E.M.A.). Also for the spatiotemporal parameters, only these three cycles were considered. All statistical analyses were based on the average of the final three selected movement cycles per task.

### Statistical analysis

To evaluate the age-related differences in UL motion, data was divided into four groups based on previous literature [9,10]: 5–7 years, 8–10 years, 11–12 years and 13–15 years. Data distribution of the spatiotemporal and kinematic parameters was evaluated using the Shapiro-Wilk test, confirming the normal distribution of all parameters except for trajectory straightness. For the normally distributed spatiotemporal parameters, mean and standard deviations were reported and one-way ANOVA with post-hoc Tukey HSD correction were computed to investigate differences between age-groups. For trajectory straightness, medians and interquartile ranges were reported and the Kruskal-Wallis test with post-hoc Mann-Whitney U tests were used. All statistical analyses were performed using SPSS (version 24.0, SPSS Inc, Chicago, IL, USA). The alpha-level was set at 0.05 for main group comparisons, with post-hoc comparisons for the six comparisons of interest (Šidák-Bonferroni correction (α’ = 1 –(1—α)^1/k^, where k represents the number of comparisons, thus α = 0.0085): 5-7y vs. 8-10y, 5-7y vs. 11-12y, 5-7y vs. 13-15y, 8-10y vs. 11-12y, 8-10y vs. 13-15y, and 11-12y vs. 13-15y. Our null hypothesis was that there is no significant difference in UL motion between the age-groups.

We calculated and reported effect sizes for main comparisons and post-hoc tests according to Cohen’s criteria: eta squared for ANOVA and Kruskal-Wallis (η^2^: small 0.01, medium 0.06, and large 0.14), Cohen’s d for post-hoc two-sample tests (d: small 0.2, medium 0.5, and large 0.8) and Cohen’s r for post-hoc Mann-Whitney tests (small 0.1, medium 0.3, and large 0.5) [27].

For the comparison of kinematic data between age-groups, a one-way ANOVA with post-hoc t-test was used with the SPM1D toolbox (version 0.4 for Matlab, available for download at http://www.spm1d.org/Downloads.html) [16]. For every joint angle, UL kinematics were compared between age-groups with the conventional univariate statistic, outputting a statistical curve (F-curve). Next, random field theory was applied to estimate the critical threshold above which only 5% of equally smoothed random data would be expected to cross (α<0.05). Whenever the statistical curve crossed the statistical threshold, a cluster was identified at the group field, leading to post-hoc t-test comparison, following the same procedure. When clusters were identified, information regarding the location (start and end points of the identified cluster) and a single p-value for each cluster was provided. The alpha-level was set at 0.05 for main group comparison. In line with the analyses of the spatiotemporal parameters, we computed post-hoc tests for the same 6 pairs with Šidák-Bonferroni correction for multiple comparisons (α<0.0085, as implemented in the toolbox). Effect sizes were computed and reported over the entire movement cycle per post-hoc comparison, according to Cohen’s d criteria (d: small 0.2, medium 0.5, and large 0.8).

## Results

### Participants

Prior to dividing the children into the four age-groups, we performed an outlier detection analysis (mean+1.96SD), resulting in the exclusion of one participant (14y, boy) based on a marked slow performance during all tasks. Given that movement duration impacts on spatiotemporal and kinematic parameters, this participant was excluded for all statistical analyses, resulting in a final group of 59 participants: 17 children aged 5–7 years (mean age (SD) 6y6m (10m), 9 boys, 14 right-handed); 16 children aged 8–10 years (9y1m±10m, 9 boys, 14 right-handed); 11 children aged 11–12 years (11y8m±9m, 9 boys, 11 right-handed); and 15 children aged 13–15 years (14y4m±12m, 8 boys, 13 right-handed).

### Spatiotemporal parameters

**Fig 1:**
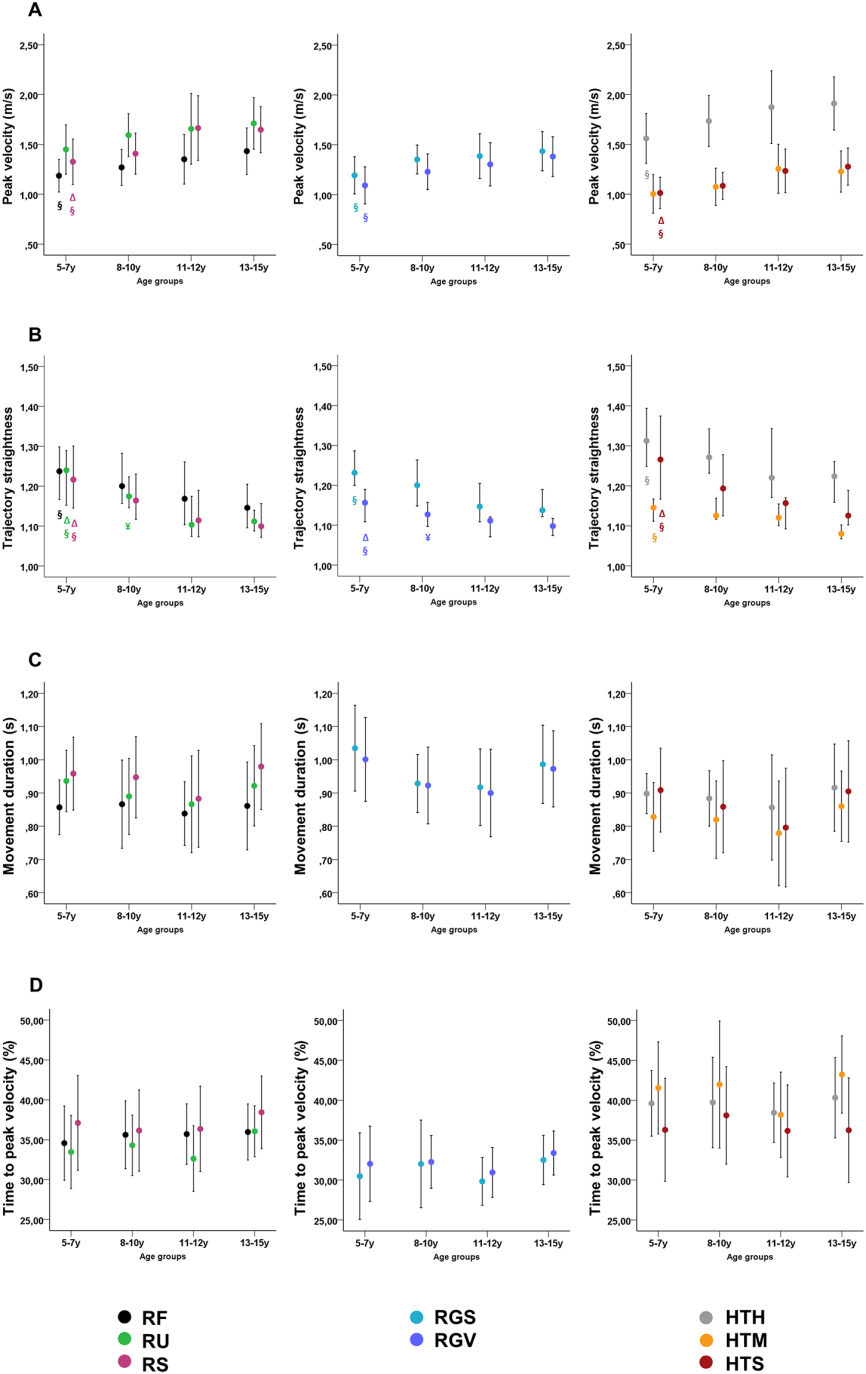
Spatiotemporal parameters per age-group for eight UL tasks (reaching, left; reach to-grasp, middle; ADL-mimicking, right): (A) peak velocity, (B) trajectory straightness, (C) movement duration, and (D) time to peak velocity. Data is shown as mean (standard deviation), except for trajectory straightness (median (first and third quartile)) RF, reaching forward; RU, reaching upwards; RS, reaching sideways; RGS, reach-to-grasp a sphere; RGV, reach-to-grasp a vertically oriented cylinder; HTH, hand-to-head; HTM, hand-to-mouth; HTS, hand-to-shoulder; y, years. Significant post-hoc differences (Bonferroni-Šidák correction α<0.0085) are shown as follows: Δ between 5-7y and 11-12y, § between 5-7y and 13-15y, and ¥ between 8-10y and 13-15y. Post-hoc analyses did not show significant differences for other between-group comparisons.

Descriptive information and statistical comparison of the spatiotemporal parameters for the whole group and per age group are summarized in Table 1. The statistical analysis of UL spatiotemporal parameters between groups showed that, with age, children progressively increased their peak velocity (all tasks, 0.0002<p<0.05) and improved their trajectory straightness (all tasks, 0.0002<p<0.01) (Fig 1). Based on post-hoc analysis, the youngest children exhibited significantly lower peak velocity than both the older groups (5-7y vs. 11-12y group, RS, p<0.004; 5-7y vs. 13-15y group, RF, RS, RGS, RGV, HTH, HTS, 0.001<p<0.007). Furthermore, the youngest group also showed a significantly less straight hand trajectory than the older groups (5-7y vs. 11-12y group, RU, RS, RGV, HTS, 0.003<p<0.007; 5-7y vs. 13-15y group, all tasks, 0.00001<p<0.006). In addition, the 8-10y group also showed less straight hand trajectory than the oldest group (8-10y vs. 13-15y, RU and HTM, 0.006<p<0.003).

**Table 1:** Descriptive and comparative statistics of the spatiotemporal parameters for the whole group and per age-group.

Tasks	Whole group (n = 59)	5-7y (n = 17)	8-10y (n = 16)	11-12y (n = 11)	13-15y (n = 15)	F/X^2^ (p-value)	Effect sizes						
							Main test (F/X^2^)	5-7y vs 8-10y	5-7y vs 11-12y	5-7y vs 13-15y	8-10y vs 11-12y	8-10y vs 13-15y	11-12y vs 13-15y
Movement duration (s) (mean (SD))													
RF	0.86 (0.11)	0.86 (0.08)	0.87 (0.13)	0.84 (0.10)	0.86 (0.13)	0.14 (0.93)	0.01	0.08	0.21	0.04	0.24	0.04	0.20
RU	0.91 (0.12)	0.94 (0.09)	0.89 (0.11)	0.87 (0.15)	0.92 (0.12)	1.00 (0.40)	0.05	0.45	0.58	0.14	0.18	0.27	0.41
RS	0.95 (0.13)	0.96 (0.11)	0.95 (0.12)	0.88 (0.15)	0.98 (0.13)	1.35 (0.26)	0.07	0.09	0.59	0.18	0.48	0.26	0.70
RGS	0.97 (0.12)	1.03 (0.13)	0.93 (0.09)	0.92 (0.12)	0.99 (0.12)	3.46 (0.02)*	0.16	0.96	0.96	0.39	0.11	0.56	0.59
RGV	0.95 (0.12)	1.00 (0.13)	0.92 (0.12)	0.90 (0.13)	0.97 (0.11)	2.05 (0.12)	0.10	0.65	0.78	0.24	0.18	0.43	0.59
HTH	0.89 (0.11)	0.90 (0.06)	0.88 (0.08)	0.86 (0.16)	0.92 (0.13)	0.68 (0.56)	0.04	0.20	0.35	0.18	0.22	0.29	0.41
HTM	0.82 (0.17)	0.83 (0.10)	0.82 (0.12)	0.78 (0.16)	0.86 (0.11)	1.00 (0.39)	0.05	0.07	0.37	0.31	0.30	0.36	0.61
HTS	0.87 (0.15)	0.91 (0.13)	0.86 (0.14)	0.80 (0.18)	0.90 (0.15)	1.63 (0.19)	0.08	0.38	0.73	0.03	0.39	0.32	0.66
Peak velocity (m/s) (mean (SD))													
RF	1.30 (0.22)	1.19 (0.16)	1.27 (0.18)	1.35 (0.25)	1.43 (0.23)	4.20 (0.01)*^§^	0.19	0.48	0.78	1.22	0.37	0.78	0.34
RU	1.59 (0.28)	1.19 (0.18)	1.35 (0.14)	1.39 (0.22)	1.43 (0.20)	2.85 (0.045)*	0.14	0.62	0.68	1.03	0.22	0.50	0.18
RS	1.49 (0.28)	1.09 (0.18)	1.23 (0.19)	1.30 (0.22)	1.38 (0.20)	7.12 (0.0004)*^Δ§^	0.28	0.37	1.20	1.40	0.94	1.10	0.06
RGS	1.33 (0.20)	1.33 (0.23)	1.41 (0.21)	1.66 (0.32)	1.65 (0.23)	5.06 (0.004)*^§^	0.22	0.96	0.94	1.27	0.18	0.48	0.23
RGV	1.24 (0.22)	1.45 (0.25)	1.59 (0.22)	1.65 (0.35)	1.71 (0.26)	6.39 (0.001)*^§^	0.26	0.75	1.04	1.50	0.37	0.81	0.38
HTH	1.75 (0.31)	1.56 (0.25)	1.73 (0.26)	1.87 (0.36)	1.91 (0.27)	4.97 (0.004)*^§^	0.21	0.69	1.00	1.35	0.44	0.67	0.12
HTM	1.13 (0.23)	1.02 (0.19)	1.07 (0.19)	1.25 (0.24)	1.23 (0.21)	5.05 (0.004)*	0.22	0.38	1.14	1.12	0.83	0.78	0.12
HTS	1.14 (0.20)	1.01 (0.16)	1.08 (0.14)	1.23 (0.22)	1.28 (0.19)	7.85 (0.0002)*^Δ§^	0.30	0.49	1.16	1.53	0.82	1.18	0.21
Time to peak velocity (%) (mean (SD))													
RF	35.42 (4.05)	34.57 (4.65)	35.61 (4.25)	35.70 (3.79)	35.97 (3.52)	0.36 (0.78)	0.02	0.23	0.27	0.34	0.02	0.09	0.07
RU	34.19 (4.05)	30.48 (5.43)	32.02 (5.49)	29.83 (3.00)	32.51 (3.09)	1.88 (0.14)	0.03	0.20	0.19	0.66	0.42	0.50	0.93
RS	37.04 (5.21)	37.11 (5.94)	36.15 (5.10)	36.35 (5.35)	38.44 (4.55)	0.57 (0.64)	0.05	0.17	0.13	0.25	0.04	0.47	0.42
RGS	31.30 (4.57)	33.46 (4.59)	34.30 (3.78)	32.63 (4.11)	36.06 (4.05)	1.04 (0.38)	0.05	0.28	0.15	0.46	0.50	0.11	0.88
RGV	32.24 (3.63)	32.04 (4.71)	32.27 (3.31)	30.96 (3.13)	33.39 (2.76)	0.98 (0.41)	0.05	0.06	0.27	0.35	0.41	0.37	0.82
HTH	39.60 (4.67)	39.61 (4.11)	39.73 (5.67)	38.43 (3.72)	40.32 (4.67)	0.40 (0.80)	0.02	0.02	0.30	0.15	0.27	0.11	0.43
HTM	41.46 (6.25)	41.56 (5.77)	41.97 (7.96)	38.17 (5.35)	43.23 (4.84)	1.48 (0.23)	0.08	0.06	0.61	0.31	0.56	0.19	0.99
HTS	36.75 (6.16)	36.29 (6.45)	38.10 (6.11)	36.17 (5.75)	36.25 (6.56)	0.34 (0.79)	0.02	0.29	0.02	0.01	0.33	0.29	0.01
Trajectory straightness (Me (IQR))													
RF	1.20 (0.11)	1.24 (0.36)	1.20 (0.12)	1.17 (0.16)	1.15 (0.11)	11.73 (0.008)*^§^	0.09	0.14	0.32	0.58	0.25	0.43	0.17
RU	1.17 (0.12)	1.24 (0.14)	1.17 (0.08)	1.10 (0.10)	1.11 (0.05)	19.29 (0.0002)*^Δ§¥^	0.16	0.33	0.51	0.69	0.36	0.50	0.02
RS	1.16 (0.12)	1.22 (0.15)	1.16 (0.27)	1.11 (0.12)	1.10 (0.08)	14.77 (0.002)*^Δ§^	0.12	0.24	0.50	0.57	0.37	0.41	0.10
RGS	1.19 (0.12)	1.23 (0.09)	1.20 (0.11)	1.15 (0.10)	1.14 (0.07)	12.19 (0.007)*^§^	0.10	0.24	0.45	0.56	0.27	0.35	0.04
RGV	1.12 (0.06)	1.16 (0.08)	1.13 (0.06)	1.10 (0.05)	1.10 (0.04)	11.47 (0.009)*^Δ^	0.09	0.28	0.55	0.40	0.41	0.31	0.05
HTH	1.26 (0.13)	1.31 (0.14)	1.27 (0.11)	1.22 (0.17)	1.22 (0.10)	11.10 (0.01)*^§^	0.09	0.28	0.45	0.49	0.25	0.35	0.01
HTM	1.12 (0.07)	1.16 (0.56)	1.12 (0.05)	1.12 (0.05)	1.08 (0.34)	12.08 (0.007)*^§¥^	0.10	0.03	0.20	0.50	0.20	0.53	0.42
HTS	1.17 (0.12)	1.27 (0.21)	1.19 (0.15)	1.16 (0.08)	1.13 (0.09)	13.76 (0.003)*^Δ§^	0.11	0.25	0.53	0.53	0.38	0.35	0.01

RF, reaching forwards; RU, reaching upwards; RS, reaching sideways; RGS, reach-to-grasp a sphere; RGV, reach-to-grasp a vertically oriented cylinder; HTH, hand-to-head; HTM, hand-to-mouth; HTS, hand-to-shoulder; Me, median; IQR, interquartile range; SD, standard deviation; s, seconds; m/s, meters per seconds; F, F-value of the ANOVA test (movement duration, peak velocity and time to peak velocity); X^2^, X^2^-value of the Kruskal-Wallis test (trajectory straightness).

* Significant differences between age-groups (α<0.05), with significant differences following Tukey HSD post-hoc testing (Šidák-Bonferroni correction of alpha (α<0.0085)): ^Δ^ between 5-7y and 11-12y, ^§^ between 5-7y and 13-15y, and ^¥^ between 8-10y and 13-15y.

Post-hoc analyses did not show significant differences for other age-group comparisons. Effect sizes for the main test (F or X^2^) and for each post-hoc comparison reported according to Cohen’s criteria: eta squared for main tests (η^2^: small 0.01, medium 0.06, and large 0.14); Cohen’s d for movement duration, peak velocity and time to peak velocity (d: small 0.2, medium 0.5, and large 0.8) and Cohen’s r for trajectory straightness (r: small 0.1, medium 0.3, and large 0.5).

For movement duration, significant age-related differences were only found for the RGS task (p = 0.02), although post-hoc analyses were not significant (0.05>p>0.99). No age-effect was found for time to peak velocity (all tasks, p>0.05).

### Kinematic waveforms

**Fig 2:**
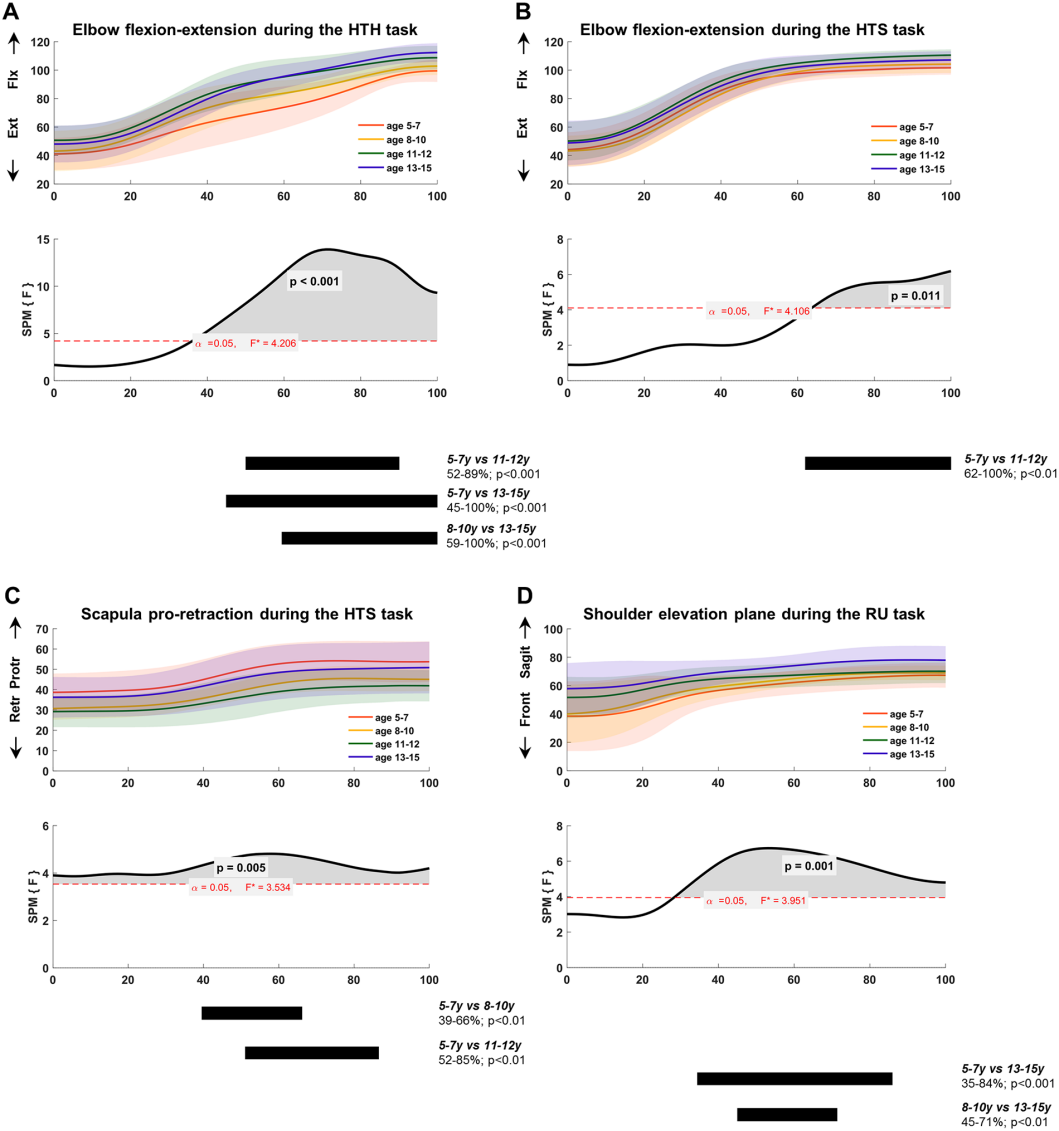
Upper limb joint kinematics for elbow flexion-extension during (A) HTH and (B) HTS, (C) scapula pro-retraction during HTS, and (D) shoulder elevation plane during RU. Top panels represent mean and standard deviation per age-group: 5-7y (orange), 8-10y (blue), 11-12y (green) and 13-15y (yellow). Bottom panels show the one-way ANOVA Statistical Parametric Mapping (SPM) output: solid black line represents the statistical curve (i.e. F-curve), red dotted line represents the statistical threshold calculated with random field theory. Significant clusters identified by SPM are shown in grey, i.e. the area where the statistical curve crosses the statistical threshold. The black bars below each graph represent the summarized SPM{t} output of the post-hoc analyses, including information regarding pair-wise comparison, cluster location, and p-values.

Based on the SPM analyses, differences in joint angle kinematics between age-groups were identified at the level of the trunk (axial rotation), scapula (medial-lateral rotation and pro-retraction), shoulder (elevation plane), and elbow (flexion-extension) (Fig 2). No age-related differences were found for the other joint angles (for details, see S1 File, with effect sizes of each comparison per joint angle and task in S2 File).

*Trunk axial rotation* differed between the four age-groups during RGS (75–100%, p<0.04), but post-hoc comparisons did not survive significance.

At the level of the *scapula*, both pro-retraction and medial-lateral rotation were significantly different between age-groups. *Scapula pro-retraction* differed during HTM and HTS (HTM, 0–10%, p<0.05; HTS, 0–100%, p<0.01). Post-hoc analyses only showed significant differences during HTS, with increased protraction in the 5-7y group compared to the 8-10y group (39–66%, p<0.01) and to the 11-12y group (52–85%, p<0.01). S*capula medial-lateral rotation* differed significantly between age-groups during movement initiation (RU, 0–10%, p<0.05; RGS, 0–13%, p<0.05), indicating that the 5-7y group used more medial rotation than the 13-15y group at movement initiation (post-hoc analysis for RGS: 0–12%, p<0.01).

We found age-related differences for *shoulder elevation plane* during two reaching tasks (RF, 10–100%, p<0.001; RU, 28–100%, p<0.01) and both reach-to-grasp tasks (RGS, 9–100%, p<0.001; RGV, 23–44%, p<0.05). Post-hoc analyses showed that the 13-15y group moved more toward the sagittal plane compared to the 5-7y group (RU: 35–84%, p<0.001) and to the 8-10y group (RF: 32–100%, p<0.001; RU: 45–71%, p<0.01 and RGS: 37–100%, p<0.001).

Lastly, *elbow flexion-extension* joint kinematics differed between age-groups during the ADL-mimicking tasks (HTH, 36–100%, p<0.001; HTS, 64–100%, p = 0.01). Post-hoc analyses indicated that the younger groups (5-7y and 8-10y) used less elbow flexion in the second half of the movement cycle compared to the older groups (11-12y and 13-15y) (Fig 2A and 2B).

## Discussion

In this study, we explored UL movement characteristics in TD children aged between 5 and 15 years to gain insights into the maturation of UL motion. We investigated the differences in both spatiotemporal and joint kinematic parameters between four age-groups, during the execution of various UL tasks. Joint kinematics were compared using SPM, which allowed us to evaluate in which part of the movement cycle the differences occurred. We were able to comprehensively describe the maturation of UL motion during a variety of tasks, including reaching, reach-to-grasp and ADL-mimicking tasks. With this detailed analysis, we provide reference values to enable direct comparison to children with UL deficits.

We found that typical UL motion becomes faster and straighter with age, and seems to reach a plateau around the age of 11-12y, and this was true for all UL tasks. This finding adds to existing literature [8,28,29], as the age-related maturation during ADL-mimicking tasks has not yet been reported. At a young age (5-7y), the fine adjustments of visual-motor coordination and motor planning required to efficiently perform UL tasks are still developing [30–32], which explains the increased movement speed and smoothness with age. Current study results further indicated that duration (except during RGS) and time to peak velocity were not age-dependent, which is in agreement with Olivier et al. [8], but in contrast to the results reported by Yan et al. [29]. However, Yan et al. [29] included 6 year old children to senior adults, and only reported a main effect of age, without specific post-hoc analyses to indicate when and how time to peak velocity changed. In contrast, Olivier et al. showed that time to peak velocity is an age-dependent parameter only when performing bimanual tasks [8].

SPM analysis was used to investigate age-related differences in UL joint kinematics during the entire movement cycle. During RGS, the youngest group (5-7y) had increased medial scapular rotation at movement initiation compared to the oldest children. This could be due to differences in the starting position, as it disappeared after the first 10% of the movement cycle. During HTS, we observed an increased scapular protraction in children aged 5-7y compared to 8-10y and 11-12y. In this task, children have to cross the body midline to reach their contralateral shoulder, and the proportionally shorter arms of younger children necessitate an increased flexibility in the scapulothoracic joint to be able to reach the contralateral body part. This would lead to a greater scapular range of motion when performing a task that requires large displacement of the scapula over the thoracic cage, such as HTS. This task appears particularly sensitive to investigate scapular motion, as we previously showed that HTS also allows discriminating scapular movement patterns between children with unilateral cerebral palsy and TD children [20]. A further study result was that younger children performed the HTH and HTS tasks with less elbow flexion compared to older children (11-12y and 13-15y), which is in line with Petuskey et al. [9]. This difference in required elbow flexion to reach the head or the contralateral shoulder might also be explained by differences in body proportions between younger and older children. Petuskey et al. additionally reported increased elbow extension in younger children during a reaching task, which was not confirmed in the present study. However, the thorough standardization of the current setup based on arm length might explain similarities in elbow movement patterns during reaching and reach-to-grasp in our study group. Lastly, we found no differences between the 11-12y and 13-15y groups for any of the joint angles and during any of the tasks, suggesting that children reach a plateau around the age of 11-12y, which is in line with previous studies [4,8].

Whilst the use of SPM is becoming more popular in UL research [20,33,34], the majority of available studies still report specific data points that are extracted from the UL joint kinematics, increasing the risk of a false positive finding (type II error). However, current study results based on SPM identified differences in UL kinematics in various parts of the movement cycle between age-groups that are not captured with standard endpoint or range of motion analyses. This discrepancy highlights the relevance of exploring the entire movement cycle to investigate UL kinematics and supports the use of SPM to investigate UL kinematic waveforms. Further validation and significance of the traditionally extracted data points (i.e. minimum and maximum joint angle, joint angle at point of task achievement) should still be investigated. These insights will potentially reduce the amount of data outputted from 3DMA and facilitate both the clinical implementation and interpretation of 3DMA in children with neurodevelopmental disorders.

Current study results showed a maturation of UL movement characteristics during reaching, reach-to-grasp, as well as ADL-mimicking tasks. Whilst we did not perform a direct comparison of performance between the tasks, results point towards a different maturation rate. Among the ADL-mimicking tasks, the hand trajectories of HTH and HTS were less straight and attained a later development plateau compared to the other UL tasks. This may be explained by the lack of visual feedback during the performance of these tasks, which may be reflected by a later maturation of visual-motor coordination if the target is out of the immediate visual field [32]. In line with these results, elbow and scapula joint kinematics also matured at an earlier age during reaching and reach-to-grasp, whilst they continued to develop during ADL-mimicking tasks. Together, this information supports the choice of reaching or reach-to-grasp tasks when comparing to children with neurodevelopmental disabilities, as age would not play a crucial role.

Some limitations of this study need to be addressed. First, our results are based on a cross-sectional study, whilst a longitudinal study design might provide additional insights in UL motion development. Furthermore, although we were able to replicate previous studies reporting the maturation effect on spatiotemporal parameters, the lack of studies reporting analyses over the entire kinematic waveform analyses impede direct comparison with our results. Therefore, the results reported in this study should be confirmed in larger sample sizes per age-group. Secondly, the age-range was selected based on common kinematic research in neurodevelopmental disorders, although this range might be too narrow to investigate the entire development of UL motion. The inclusion of younger children would provide further insights on the early maturation of UL motion, whereas extending the age-range up to 18 years would confirm the maturation plateau. However, UL-3DMA in children below 5 years old is challenging, as the marker placement becomes increasingly difficult on smaller arms and hands. Other approaches, e.g. sensor-based techniques, may be more suitable to investigate UL motion in this younger group [35]. Additionally, these sensor-based approaches would permit an ‘out of the lab’ UL motion analysis, moving toward a measure of daily live UL function. In this line, the main advantage of 3DMA is that it offers a valid, quantitative, and objective tool to comprehensively evaluate movement patterns and to identify the specific UL problems, consequently, contributing to guiding interventions on the UL (e.g. botulinum toxin injections [36], muscle lengthening or other orthopedic approaches [37]. However, this technique may result in a large time investment with high associated costs, due to the expensive equipment and the required experience of the clinicians. Consequently, there is a necessity of further research to elucidate when and for who this tool can serve as an added value, both within a clinical as well as a socio-economic perspective. For example, by evaluating when and for who a 3DMA may be truly beneficial compared to the traditionally used clinical scales, health resources may be better allocated, resulting in the adequate time and cost investment for the patient and the clinicians.

A SPM related limitation for post-hoc testing should also be addressed for caution interpretation of the results. At present, only an adjustment of alpha threshold following Bonferroni and Šidák-Bonferroni procedures is available in the SPM1d toolbox. Other less stringent and conservative post-hoc procedures will warrant increased statistical power and a more accurate control of type I error [38]. Lastly, the influence of biological maturation aspects (i.e. skeletal age or growth spurts) and/or the impact of specific UL motor skills the participants may have acquired during their childhood, e.g. playing music instruments or sports, were not considered. Gaining insights in the role of these motor skills and different aspects of biological maturation in UL motion development may contribute to further understanding whether these covariates should be considered in further investigations.

Current study results provide a reference database of typical development of UL motion. Reporting normative values and age-related differences will contribute to an increased knowledge of the typical maturation of UL movement characteristics, and consequently an improved interpretation of UL deficits in children. This is crucial to efficiently allocate therapeutic resources in UL rehabilitation. Lastly, although these results have provided information regarding the effect of age on spatiotemporal parameters and kinematics during a variety of UL tasks in TD children, the potential age-dependency of UL motion in children with neurodevelopmental disorders needs to be further explored.

## Conclusion

UL motion characteristics showed age-related changes in children between 5–15 years old. With age, UL movements become faster and straighter. Furthermore, age-related and task-dependent changes were also reported for UL kinematics at the level of the scapular medial-lateral rotation and pro-retraction, shoulder elevation plane and elbow flexion-extension angles. Current study results highlight the importance of investigating the entire UL motion to decrease the false-positive rate, as demonstrated with SPM. The normative values may be valuable to gain insights into typical UL maturation and to compare with children with UL deficits, which is fundamental for rehabilitation programs.

## Supporting information

S1 File Figs (S1-S8) representing the upper limb movement patterns of all four age-groups during (S1) reaching forward, (S2) reaching upward, (S3) reaching sideways, (S4) reach-to-grasp a sphere, (S5) reach-to-grasp a vertically oriented cylinder, (S6) hand-to-mouth, (S7) hand-to-head, (S8) hand-to-shoulder. Joints are presented in rows, from top to bottom: trunk, scapula, shoulder, elbow, and wrist. Data is shown as mean (bold line) and standard deviation (translucent area) for every age-group: 5-7y (red), 8-10y (orange), 11-12y (green) and 13-15y (blue).
(PDF)

S2 File Figs (S9-S16) representing the effect sizes (Cohen’s d) of the pair-wise post-hoc comparisons during (S9) reaching forward, (S10) reaching upward, (S11) reaching sideways, (S12) reach-to-grasp a sphere, (S13) reach-to-grasp a vertically oriented cylinder, (S14) hand-to-mouth, (S15) hand-to-head, (S16) hand-to-shoulder. Joints are presented in rows, from top to bottom: trunk, scapula, shoulder, elbow, and wrist. Data is shown as effect sizes for every pair-wise post-hoc comparison after ANOVA test: 5-7y vs 8-10y (brown), 5-7y vs 11-12y (red), 5-7y vs 13-15y (purple), 8-10y vs 11-12y (blue), 8-10y vs 13-15y (green), and 11-12y vs 13-15y (yellow). Effect sizes are reported according to Cohen’s d criteria: small 0.2, medium 0.5, and large 0.8 over the movement cycle.
(PDF)

## References

[1] Chen C-M, Chen C-Y, Wu KP, Chen C-L, Hsu H-C, Lo S-K. Motor Factors Associated with Health-Related Quality-of-Life in Ambulatory Children with Cerebral Palsy. American Journal of Physical Medicine & Rehabilitation. 2011;90: 940–947. 21904192 10.1097/PHM.0b013e3182240d54

[2] Janssen MMHP, Bergsma A, Geurts ACH, de Groot IJM. Patterns of decline in upper limb function of boys and men with DMD: an international survey. Journal of Neurology. 2014;261: 1269–1288. doi: 10.1007/s00415-014-7316-9 24687893 10.1007/s00415-014-7316-9

[3] Schneiberg S, Sveistrup H, McFadyen B, McKinley P, Levin MF. The development of coordination for reach-to-grasp movements in children. Experimental Brain Research. 2002;146: 142–154. doi: 10.1007/s00221-002-1156-z 12195516 10.1007/s00221-002-1156-z

[4] Gilliaux M, Dierckx F, Vanden Berghe L, Lejeune TM, Sapin J, Dehez B, et al Age Effects on Upper Limb Kinematics Assessed by the REAplan Robot in Healthy School-Aged Children. Annals of Biomedical Engineering. 2015;43: 1123–1131. doi: 10.1007/s10439-014-1189-z 25413362 10.1007/s10439-014-1189-z

[5] Berthier NE, Keen R. Development of reaching in infancy. Experimental Brain Research. 2006;169: 507–518. doi: 10.1007/s00221-005-0169-9 16341854 10.1007/s00221-005-0169-9

[6] Konczak J, Dichgans J. The development toward stereotypic arm kinematics during reaching in the first 3 years of life. Experimental Brain Research. Springer-Verlag; 1997;117: 346–354. doi: 10.1007/s002210050228 10.1007/s002210050228 9419079

[7] Kuhtz-Buschbeck JP, Stolze H, Jöhnk K, Boczek-Funcke A, Illert M. Development of prehension movements in children: a kinematic study. Experimental Brain Research. 1998;122: 424–432. doi: 10.1007/s002210050530 9827861 10.1007/s002210050530

[8] Olivier I, Hay L, Bard C, Fleury M. Age-related differences in the reaching and grasping coordination in children: unimanual and bimanual tasks. Experimental Brain Research. 2007;179: 17–27. doi: 10.1007/s00221-006-0762-6 17091289 10.1007/s00221-006-0762-6

[9] Petuskey K, Bagley A, Abdala E, James MA, Rab G. Upper extremity kinematics during functional activities: Three-dimensional studies in a normal pediatric population. Gait & Posture. 2007;25: 573–579. doi: 10.1016/j.gaitpost.2006.06.006 16875821 10.1016/j.gaitpost.2006.06.006

[10] Beaudette B, Chester VL. Upper Extremity Kinematics in Pediatric and Young Adult Populations during Activities of Daily Living. Journal of Medical and Biological Engineering. 2014;34: 448–454.

[11] Butler EE, Ladd AL, Louie SA, LaMont LE, Wong W, Rose J. Three-dimensional kinematics of the upper limb during a Reach and Grasp Cycle for children. Gait & Posture. Elsevier B.V.; 2010;32: 72–77. doi: 10.1016/j.gaitpost.2010.03.011 20378351 10.1016/j.gaitpost.2010.03.011

[12] Jaspers E, Desloovere K, Bruyninckx H, Klingels K, Molenaers G, Aertbeliën E, et al Three-dimensional upper limb movement characteristics in children with hemiplegic cerebral palsy and typically developing children. Research in Developmental Disabilities. 2011;32: 2283–2294. doi: 10.1016/j.ridd.2011.07.038 21862283 10.1016/j.ridd.2011.07.038

[13] Mahon J, Malone A, Kiernan D, Meldrum D. Reliability of 3D upper limb motion analysis in children with obstetric brachial plexus palsy. Physiological Measurement. 2017;38: 524–538. doi: 10.1088/1361-6579/aa5c13 28140349 10.1088/1361-6579/aa5c13

[14] De Baets L, Van Deun S, Monari D, Jaspers E. Three-dimensional kinematics of the scapula and trunk, and associated scapular muscle timing in individuals with stroke. Human Movement Science. 2016;48: 82–90. doi: 10.1016/j.humov.2016.04.009 27155342 10.1016/j.humov.2016.04.009

[15] Pataky TC, Vanrenterghem J, Robinson MA. The probability of false positives in zero-dimensional analyses of one-dimensional kinematic, force and EMG trajectories. Journal of Biomechanics. 2016;49: 1468–1476. doi: 10.1016/j.jbiomech.2016.03.032 27067363 10.1016/j.jbiomech.2016.03.032

[16] Pataky TC. Generalized n-dimensional biomechanical field analysis using statistical parametric mapping. Journal of Biomechanics. Elsevier; 2010;43: 1976–1982. doi: 10.1016/j.jbiomech.2010.03.008 20434726 10.1016/j.jbiomech.2010.03.008

[17] Brochard S, Lempereur M, Mao L, Rémy-Néris O. The role of the scapulo-thoracic and gleno-humeral joints in upper-limb motion in children with hemiplegic cerebral palsy. Clinical Biomechanics. 2012;27: 652–660. doi: 10.1016/j.clinbiomech.2012.04.001 22560625 10.1016/j.clinbiomech.2012.04.001

[18] Jaspers E, Feys H, Bruyninckx H, Klingels K, Molenaers G, Desloovere K. The Arm Profile Score: A new summary index to assess upper limb movement pathology. Gait & Posture. Elsevier B.V.; 2011;34: 227–233. doi: 10.1016/j.gaitpost.2011.05.003 21641224 10.1016/j.gaitpost.2011.05.003

[19] Mailleux L, Jaspers E, Ortibus E, Simon-Martinez C, Desloovere K, Molenaers G, et al Clinical assessment and three-dimensional movement analysis: An integrated approach for upper limb evaluation in children with unilateral cerebral palsy. Kellermayer MS, editor. PLOS ONE. 2017;12: e0180196 doi: 10.1371/journal.pone.0180196 28671953 10.1371/journal.pone.0180196 PMC5495347

[20] Simon-Martinez C, Jaspers E, Mailleux L, Desloovere K, Vanrenterghem J, Ortibus E, et al Negative Influence of Motor Impairments on Upper Limb Movement Patterns in Children with Unilateral Cerebral Palsy. A Statistical Parametric Mapping Study. Frontiers in Human Neuroscience. 2017;11: 482 doi: 10.3389/fnhum.2017.00482 29051729 10.3389/fnhum.2017.00482 PMC5633911

[21] Coluccini M, Maini ES, Martelloni C, Sgandurra G, Cioni G. Kinematic characterization of functional reach to grasp in normal and in motor disabled children. Gait & Posture. Elsevier; 2007;25: 493–501. doi: 10.1016/J.GAITPOST.2006.12.015 17270446 10.1016/j.gaitpost.2006.12.015

[22] Wu G, van der Helm FCT, (DirkJan) Veeger HEJ, Makhsous M, Van Roy P, Anglin C, et al ISB recommendation on definitions of joint coordinate systems of various joints for the reporting of human joint motion—Part II: shoulder, elbow, wrist and hand. Journal of Biomechanics. 2005;38: 981–992. doi: 10.1016/j.jbiomech.2004.05.042 15844264 10.1016/j.jbiomech.2004.05.042

[23] Jaspers E, Feys H, Bruyninckx H, Harlaar J, Molenaers G, Desloovere K. Upper limb kinematics: Development and reliability of a clinical protocol for children. Gait & Posture. 2011;33: 279–285. doi: 10.1016/j.gaitpost.2010.11.021 21196120 10.1016/j.gaitpost.2010.11.021

[24] Jaspers E, Desloovere K, Bruyninckx H, Molenaers G, Klingels K, Feys H. Review of quantitative measurements of upper limb movements in hemiplegic cerebral palsy. Gait & Posture. 2009;30: 395–404. doi: 10.1016/j.gaitpost.2009.07.110 19679479 10.1016/j.gaitpost.2009.07.110

[25] Woltring HJ. Smoothing and differentiation techniques applied to 3-D data Franklin I. Allard P, Stokes I, Blanchi J, editors. Three-dimensional analysis of human movement. Champaign, IL: Human Kinetics; 1995.

[26] Meskers CGM, van der Helm FCT, Rozendaal LA, Rozing PM. In vivo estimation of the glenohumeral joint rotation center from scapular bony landmarks by linear regression. Journal of Biomechanics. 1997;31: 93–96. doi: 10.1016/S0021-9290(97)00101-2 10.1016/s0021-9290(97)00101-2 9596544

[27] Cohen J. Statistical power analysis for the behavioral sciences. 2nd Editio Hillsdale, NJ Elsevier Science; 1988.

[28] Chiappedi M, Togni R, De Bernardi E, Baschenis IMC, Battezzato S, Balottin U, et al Arm trajectories and writing strategy in healthy children. BMC Pediatrics. BioMed Central; 2012;12: 695 doi: 10.1186/1471-2431-12-173 23134839 10.1186/1471-2431-12-173 PMC3507863

[29] Yan JH, Thomas JR, Stelmach GE, Thomas KT. Developmental Features of Rapid Aiming Arm Movements Across the Lifespan. Journal of Motor Behavior. 2000;32: 121–140. doi: 10.1080/00222890009601365 11005944 10.1080/00222890009601365

[30] van Donkelaar P, Franks IM. Preprogramming vs. on-line control in simple movement sequences. Acta Psychologica. 1991;77: 1–19. doi: 10.1016/0001-6918(91)90061-4 1950633 10.1016/0001-6918(91)90061-4

[31] Hay L. Accuracy of Children on an Open-Loop Pointing Task. Perceptual and Motor Skills. 1978;47: 1079–1082. doi: 10.2466/pms.1978.47.3f.1079 745878 10.2466/pms.1978.47.3f.1079

[32] Jongbloed-Pereboom M, Nijhuis-van der Sanden MWG, Saraber-Schiphorst N, Crajé C, Steenbergen B. Anticipatory action planning increases from 3 to 10years of age in typically developing children. Journal of Experimental Child Psychology. 2013;114: 295–305. doi: 10.1016/j.jecp.2012.08.008 23026314 10.1016/j.jecp.2012.08.008

[33] Santos GL, Russo TL, Nieuwenhuys A, Monari D, Desloovere K. Kinematic Analysis of a Drinking Task in Chronic Hemiparetic Patients Using Features Analysis and Statistical Parametric Mapping. Archives of Physical Medicine and Rehabilitation. The American Congress of Rehabilitation Medicine; 2018;99: 501–511.e4. doi: 10.1016/j.apmr.2017.08.479 28939425 10.1016/j.apmr.2017.08.479

[34] Li X, Santago AC, Vidt ME, Saul KR. Analysis of effects of loading and postural demands on upper limb reaching in older adults using statistical parametric mapping. Journal of Biomechanics. 2016;49: 2806–2816. doi: 10.1016/j.jbiomech.2016.06.018 27435566 10.1016/j.jbiomech.2016.06.018 PMC5056136

[35] Cahill-Rowley K, Rose J. Temporal–spatial reach parameters derived from inertial sensors: Comparison to 3D marker-based motion capture. Journal of Biomechanics. 2017;52: 11–16. doi: 10.1016/j.jbiomech.2016.10.031 28010947 10.1016/j.jbiomech.2016.10.031

[36] Mackey A, Stinear C, Stott S, Byblow WD. Upper Limb Function and Cortical Organization in Youth with Unilateral Cerebral Palsy. Frontiers in Neurology. 2014;5: 117 doi: 10.3389/fneur.2014.00117 25071705 10.3389/fneur.2014.00117 PMC4082181

[37] DeLuca PA, Davis RB, Õunpuu S, Rose S, Sirkin R. Alterations in Surgical Decision Making in Patients with Cerebral Palsy Based on Three-Dimensional Gait Analysis. Journal of Pediatric Orthopaedics. 1997;17: 608–614. doi: 10.1097/01241398-199709000-00007 9591998 10.1097/00004694-199709000-00007

[38] Kim H-Y. Statistical notes for clinical researchers: post-hoc multiple comparisons. Restorative Dentistry & Endodontics. Korean Academy of Conservative Dentistry; 2015;40: 172–176. doi: 10.5395/rde.2015.40.2.172 25984481 10.5395/rde.2015.40.2.172 PMC4432262

