# Supplementary material for: Age-related changes in upper limb motion during typical development
Source: PLoS One. 2018 Jun 6;13(6):e0198524. doi: 10.1371/journal.pone.0198524 (PMC5991355; doi:10.1371/journal.pone.0198524)

# Fig S1. Reaching forwards (RF)

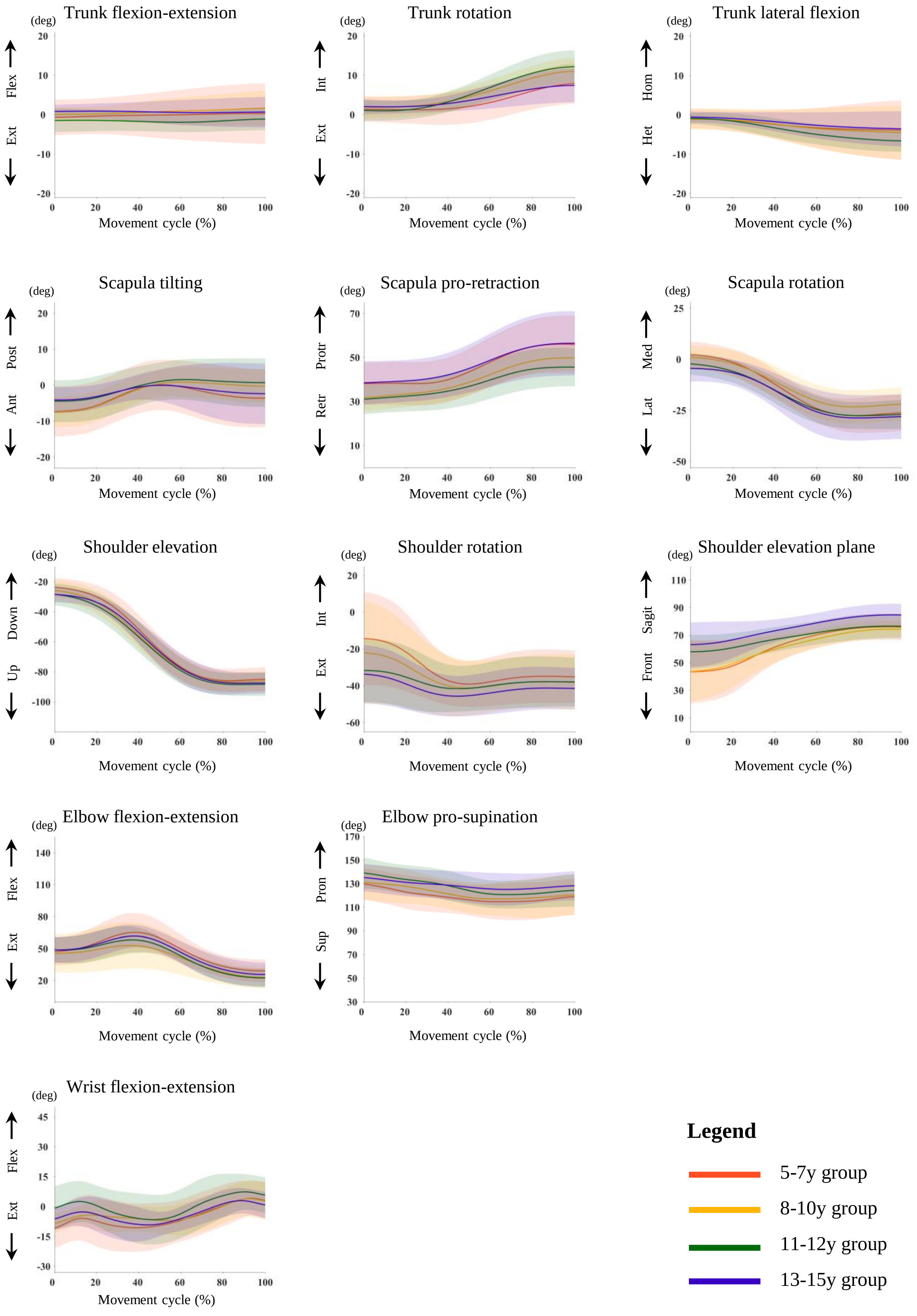

# Fig S2. Reaching upwards (RU)

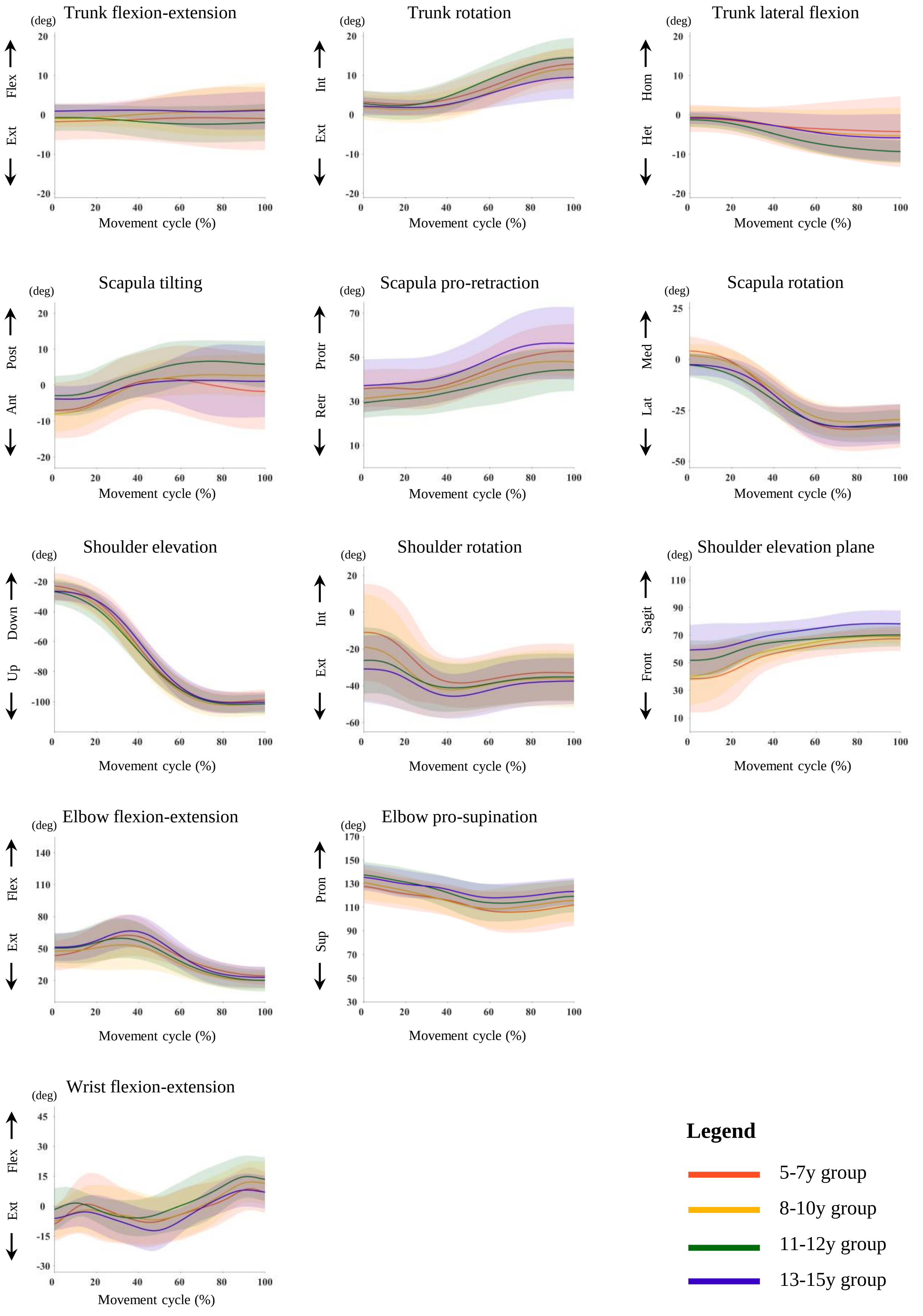

# Fig S3. Reaching sideways (RS)

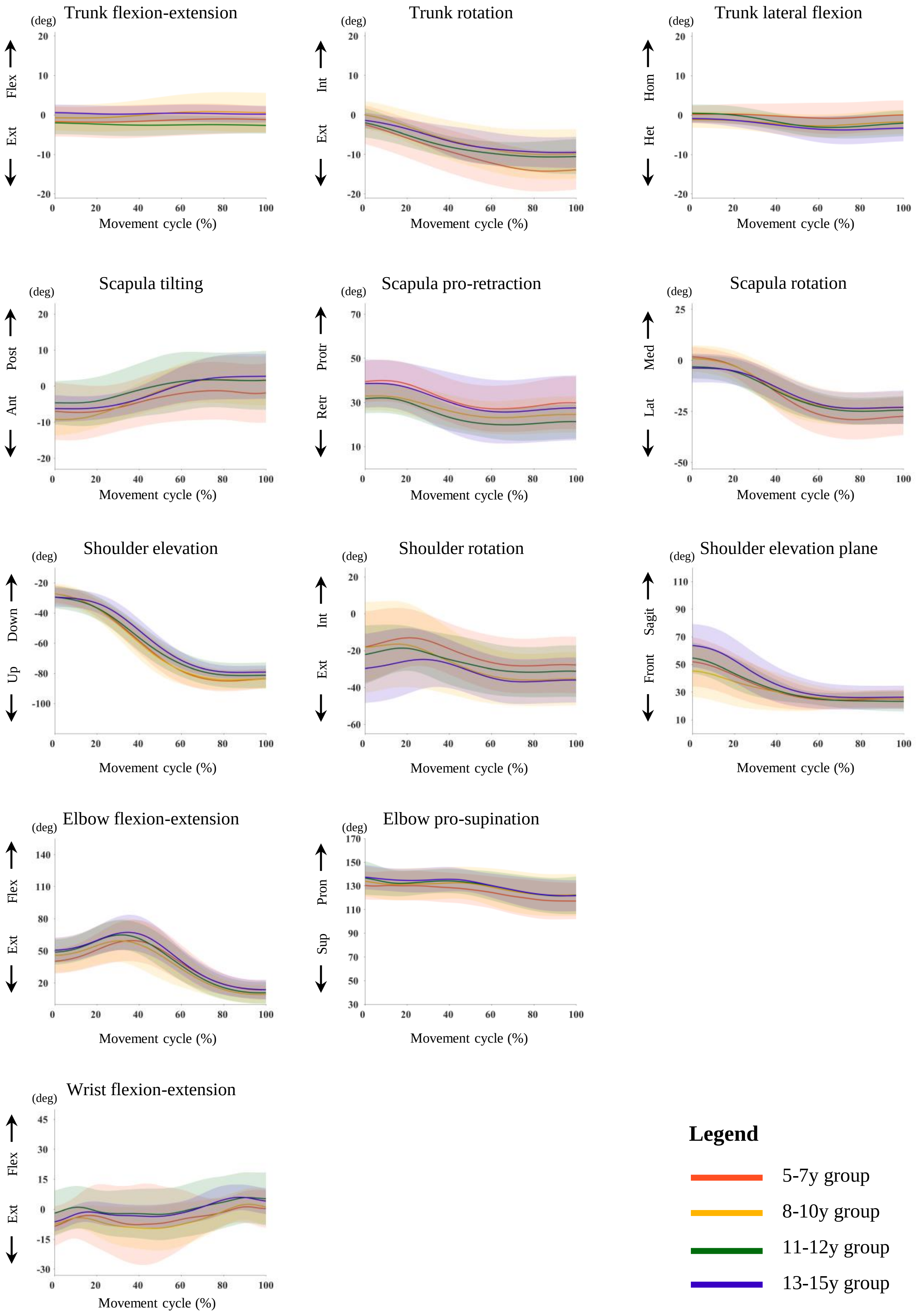

# Fig S4. Reach to grasp a sphere (RGS)

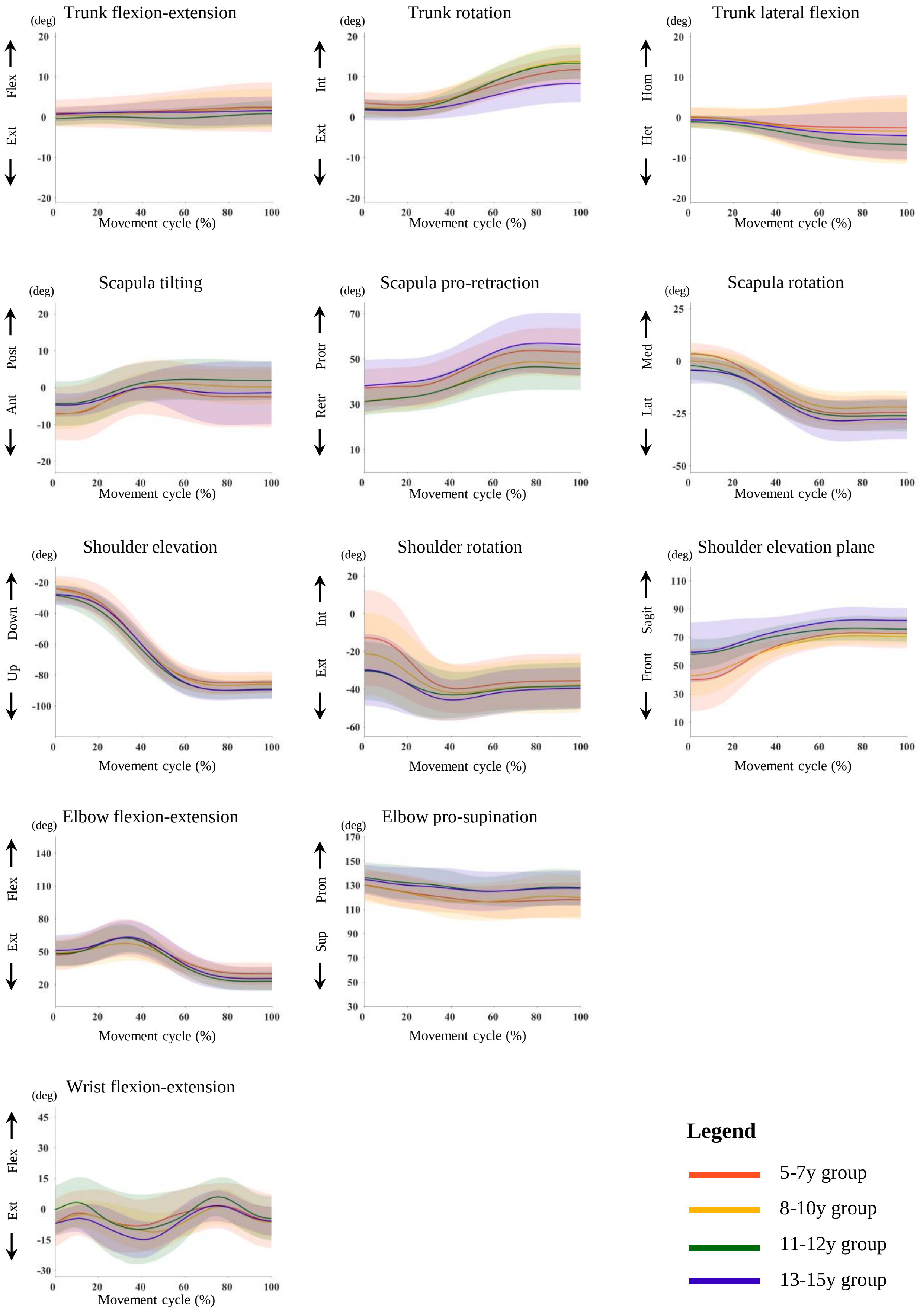

# Fig S5. Reach to grasp a vertically oriented cylinder (RGV)

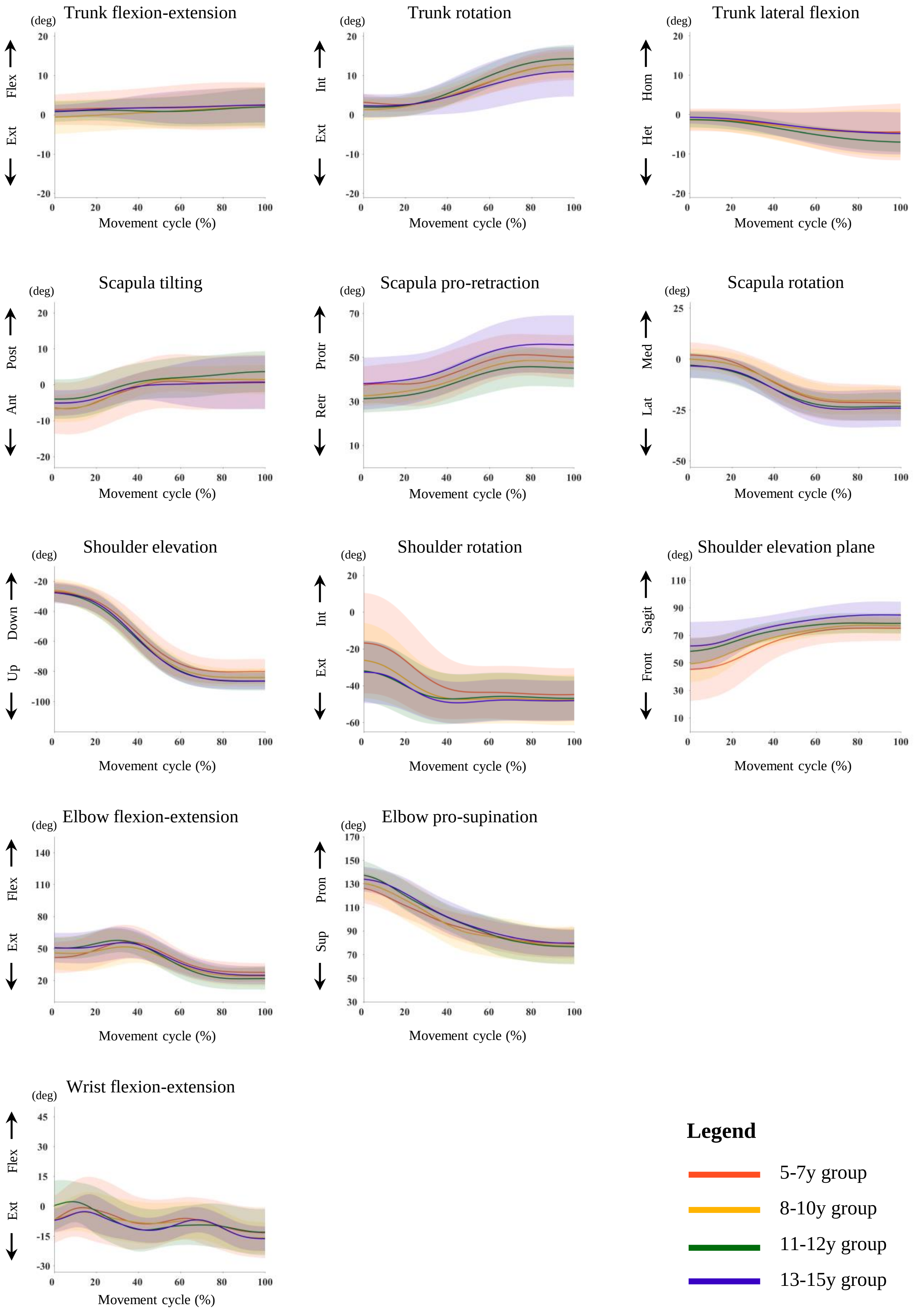

# Fig S6. Hand to head (HTH)

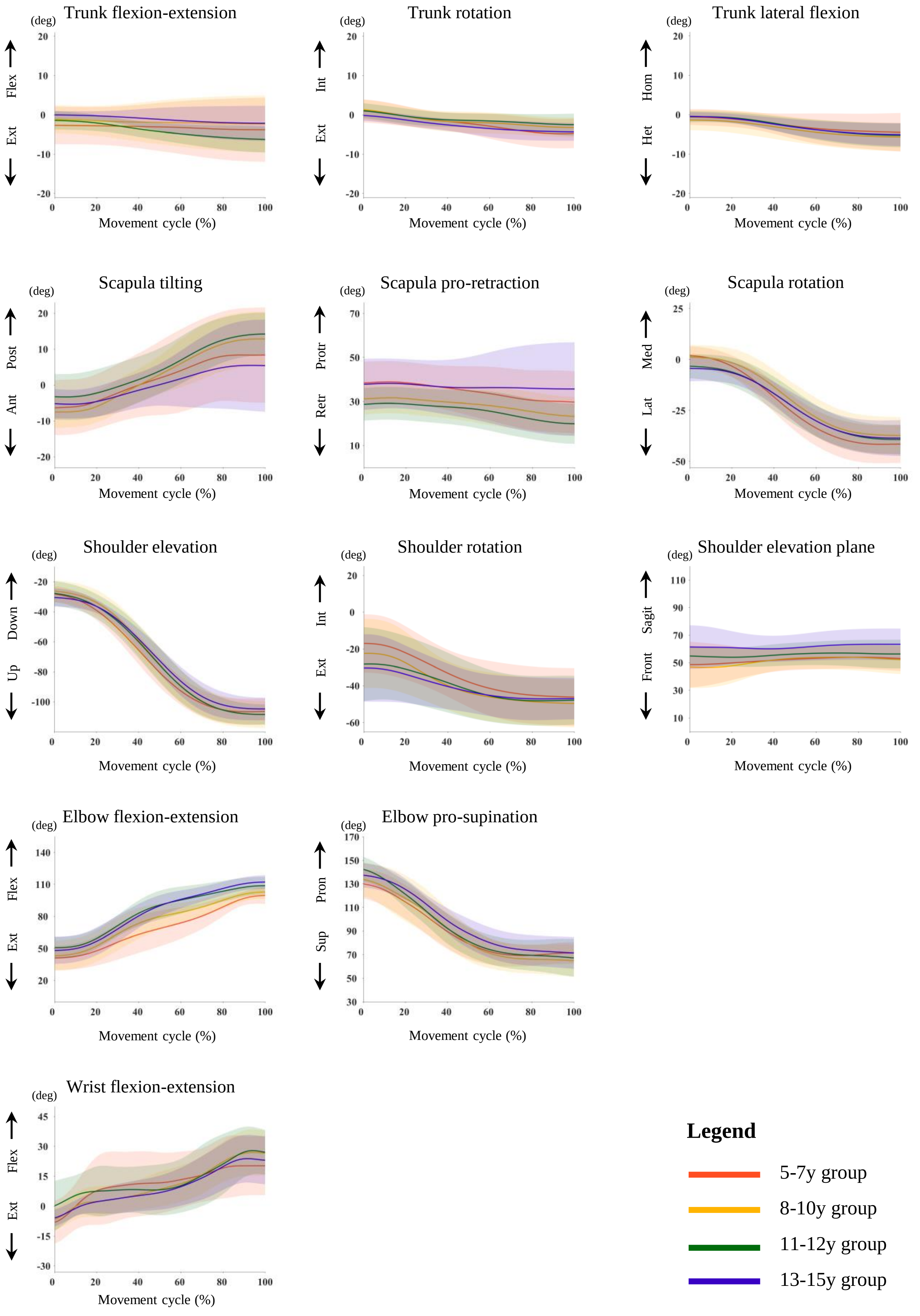

# Fig S7. Hand to mouth (HTM)

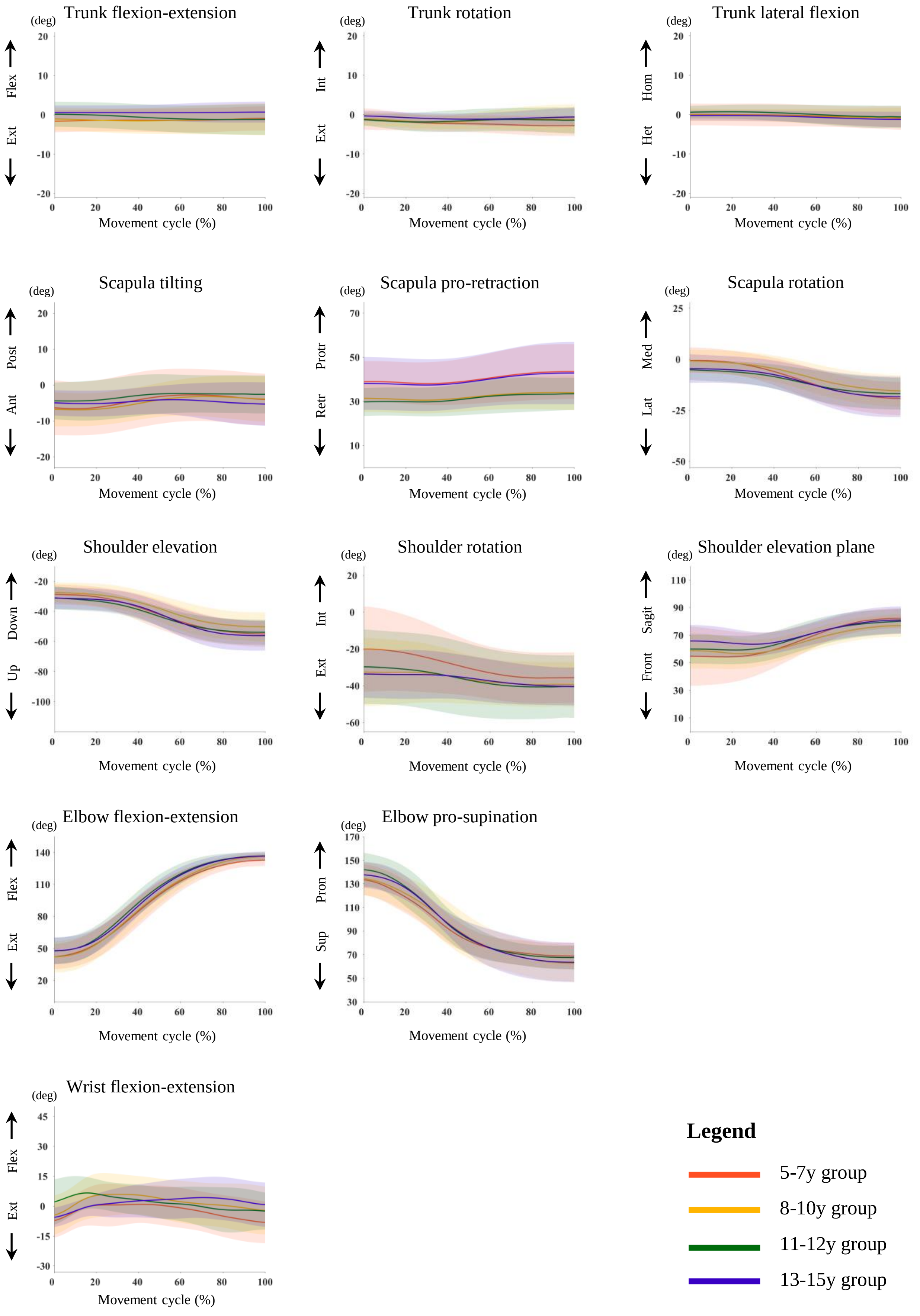

# Fig S8. Hand to shoulder (HTS)

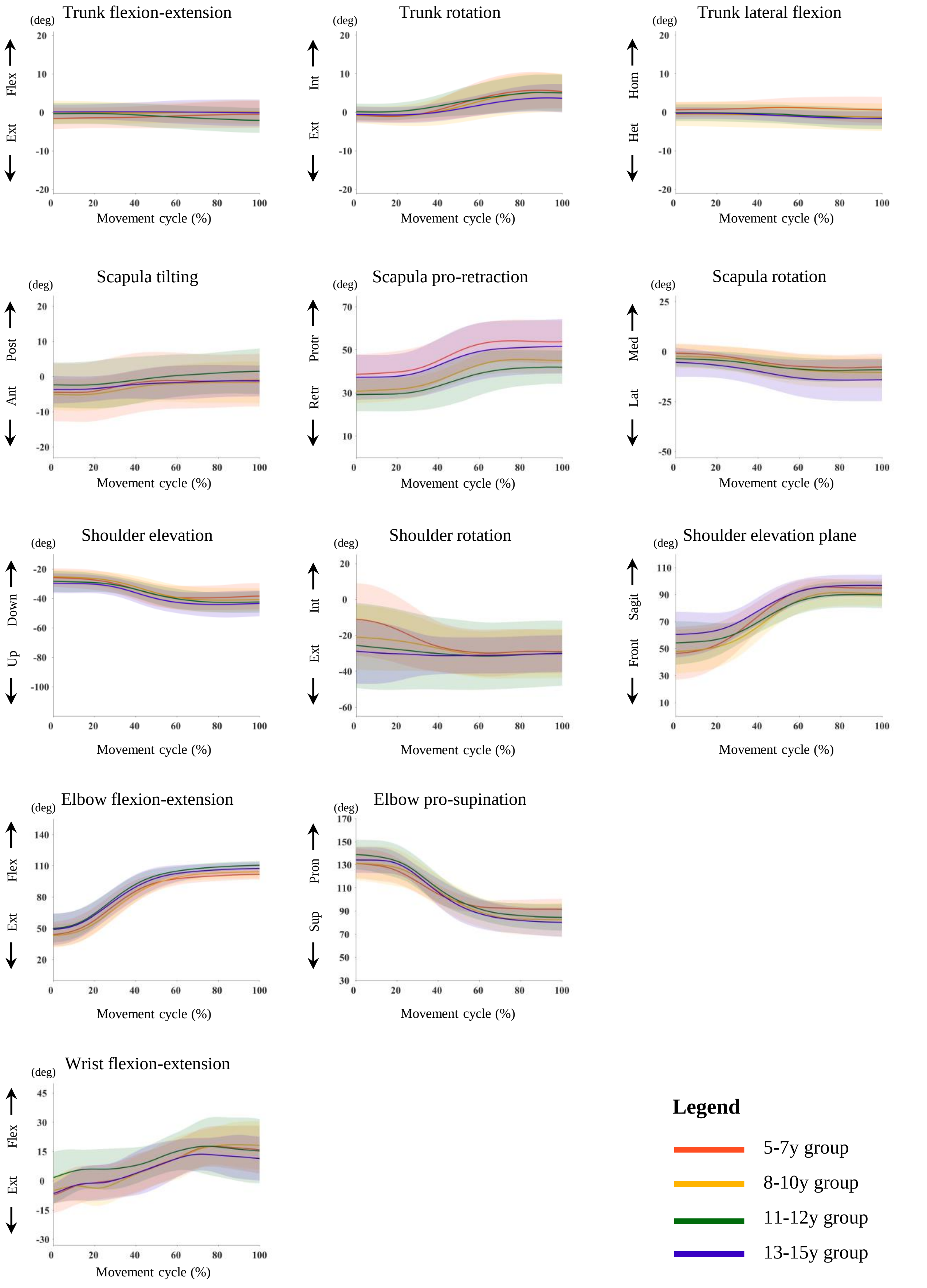

Supplement: S1 File — Joints are presented in rows, from top to bottom: trunk, scapula, shoulder, elbow, and wrist. Data is shown as mean (bold line) and standard deviation (translucent area) for every age-group: 5-7y (red), 8-10y (orange), 11-12y (green) and 13-15y (blue). (PDF) [file pone.0198524.s001.pdf]
