# Supplementary material for: Age-related changes in upper limb motion during typical development
Source: PLoS One. 2018 Jun 6;13(6):e0198524. doi: 10.1371/journal.pone.0198524 (PMC5991355; doi:10.1371/journal.pone.0198524)

Fig S9. Reaching forwards (RF)

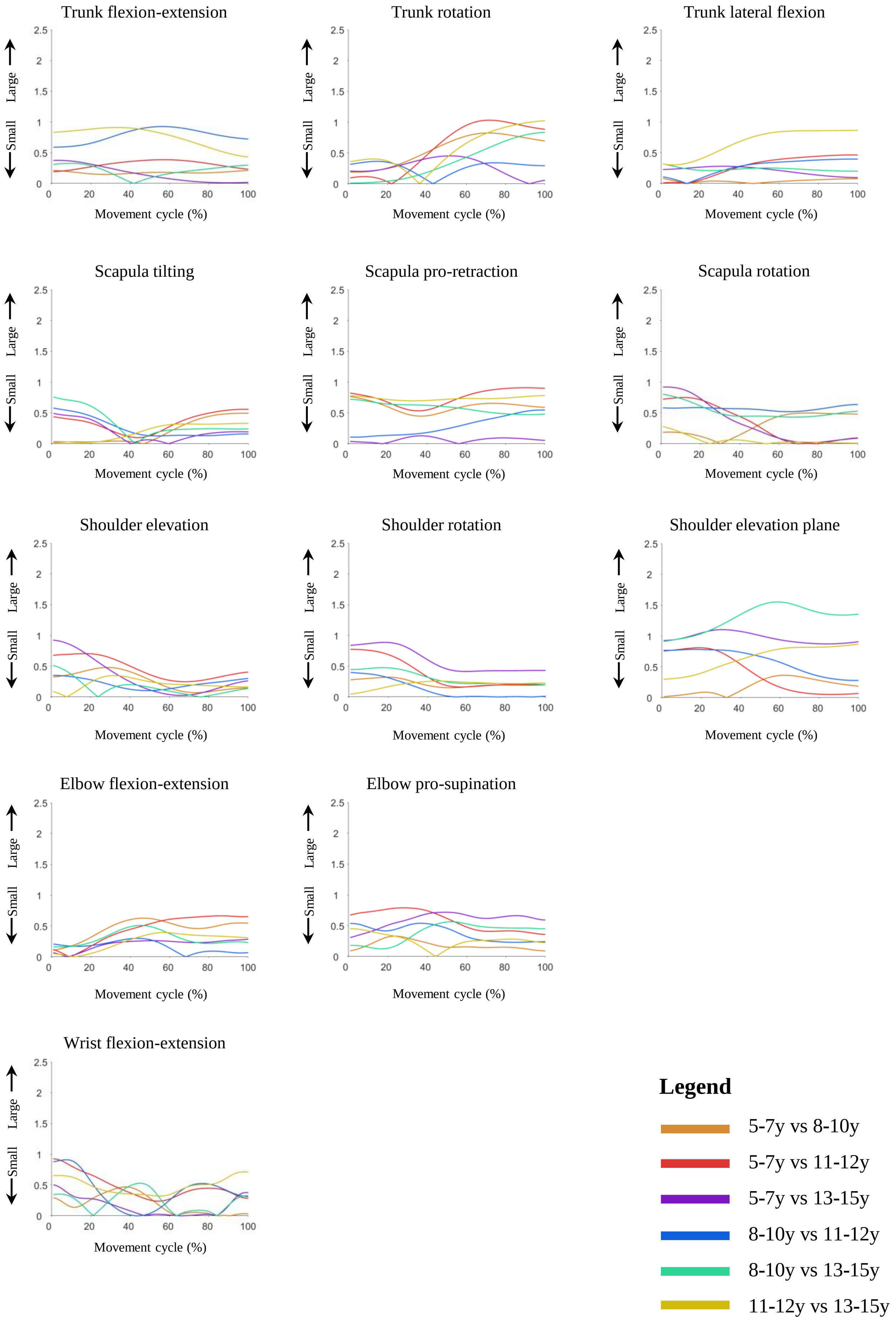

Fig S10. Reaching upwards (RU)

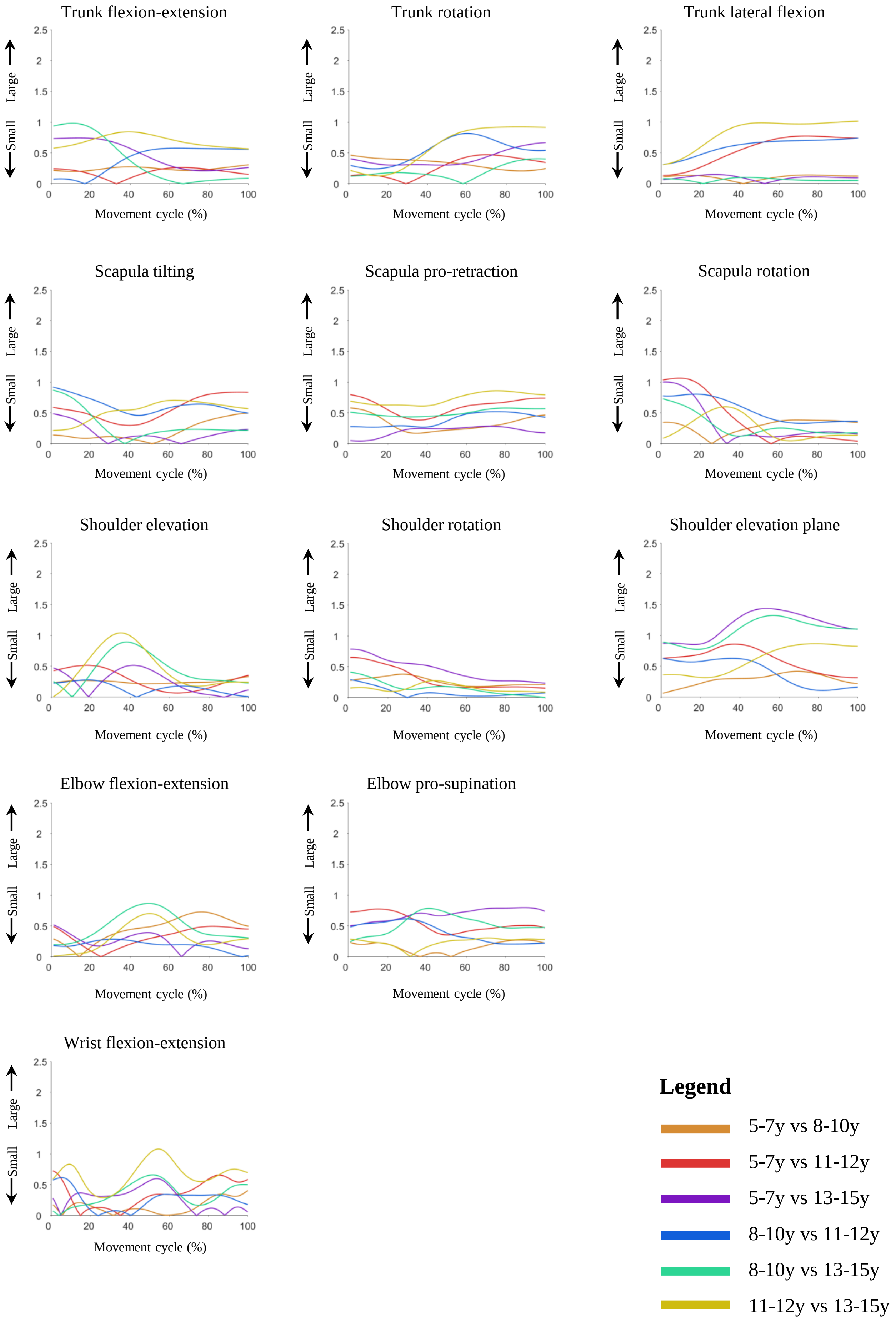

Fig S11. Reaching sideways (RS)

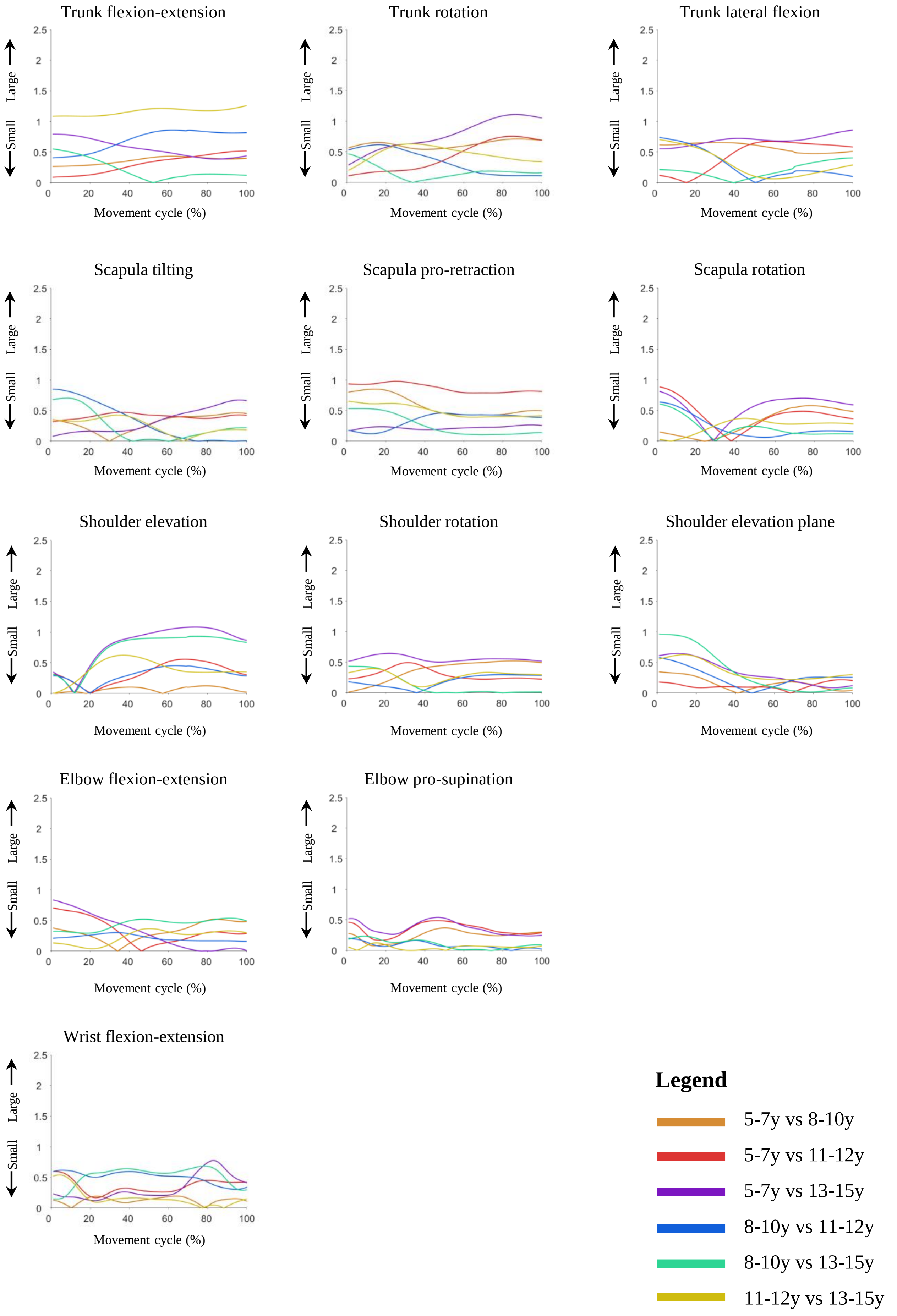

Fig S12. Reach-to-grasp a sphere (RGS)

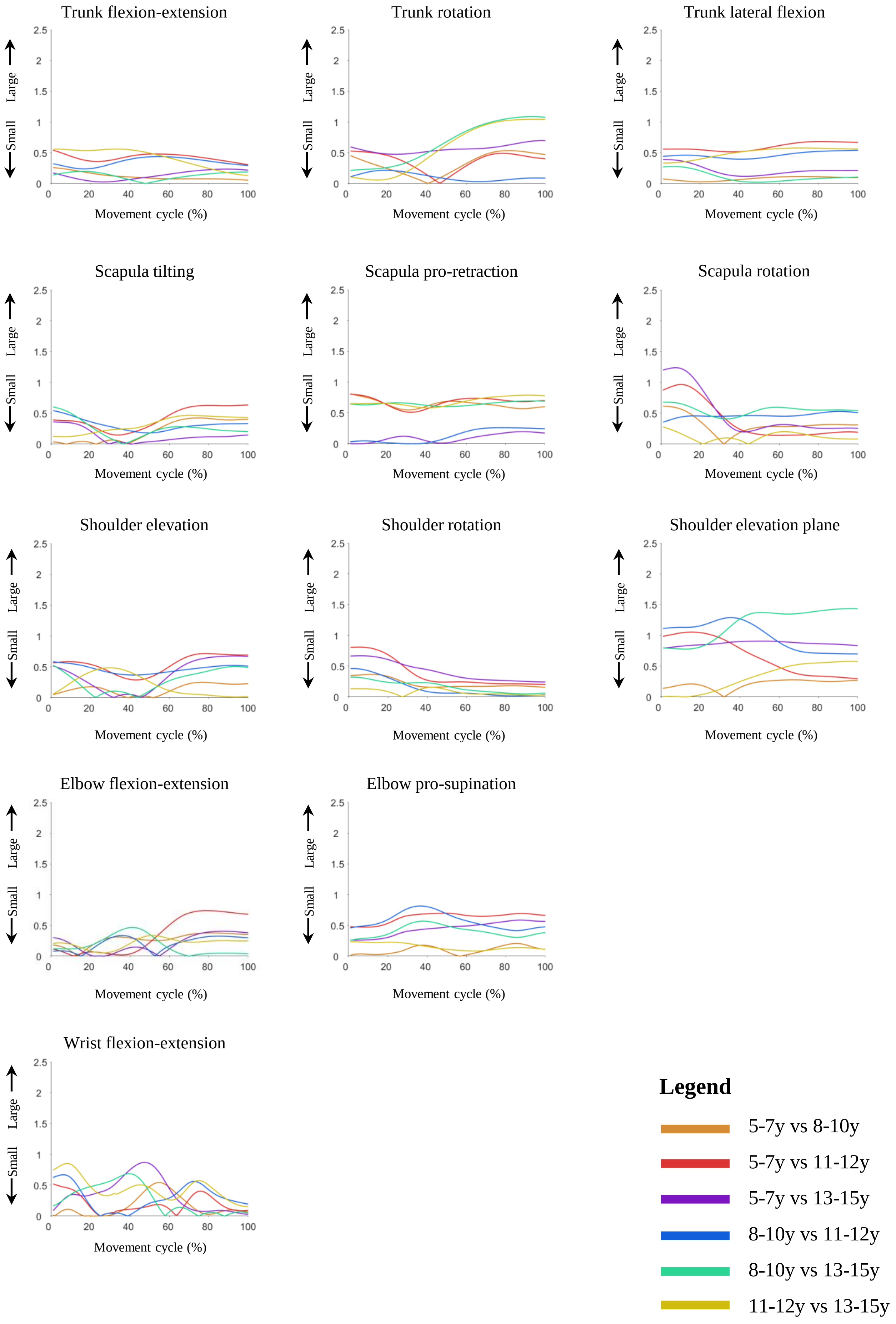

Fig S13. Reach-to-grasp a vertically oriented cylinder (RGV)

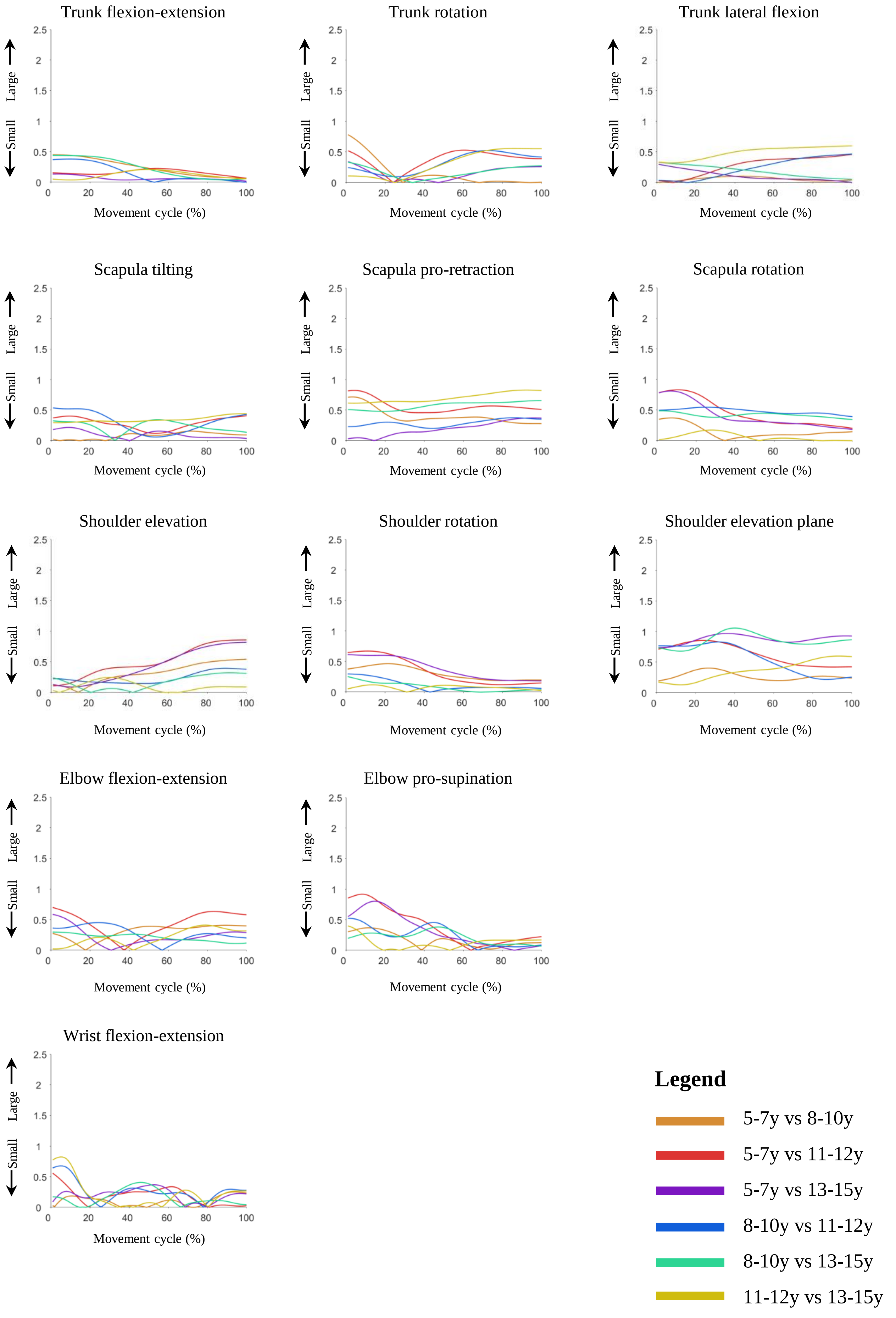

Fig S14. Hand to Head (HTH)

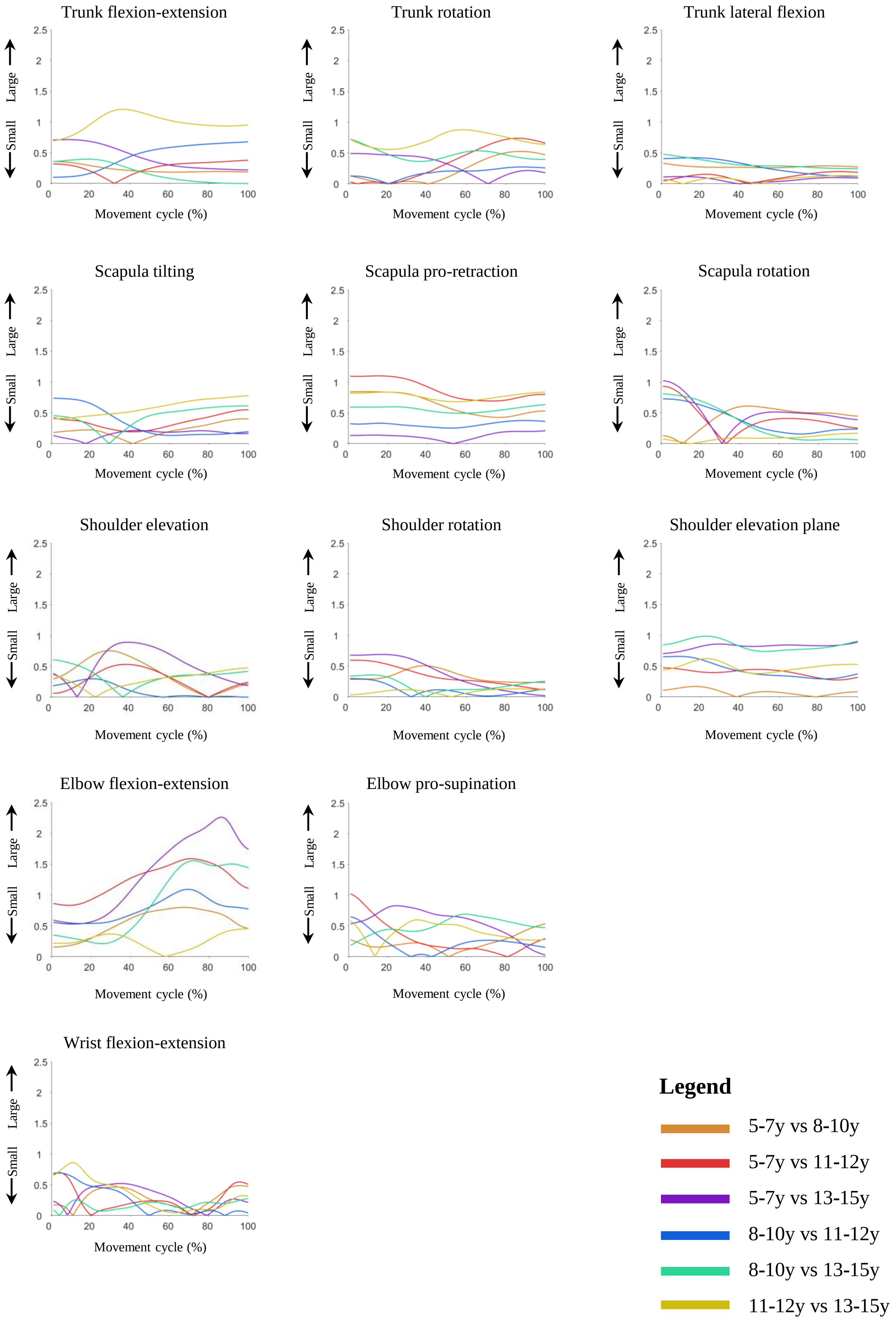

Fig S15. Hand to Mouth (HTM)

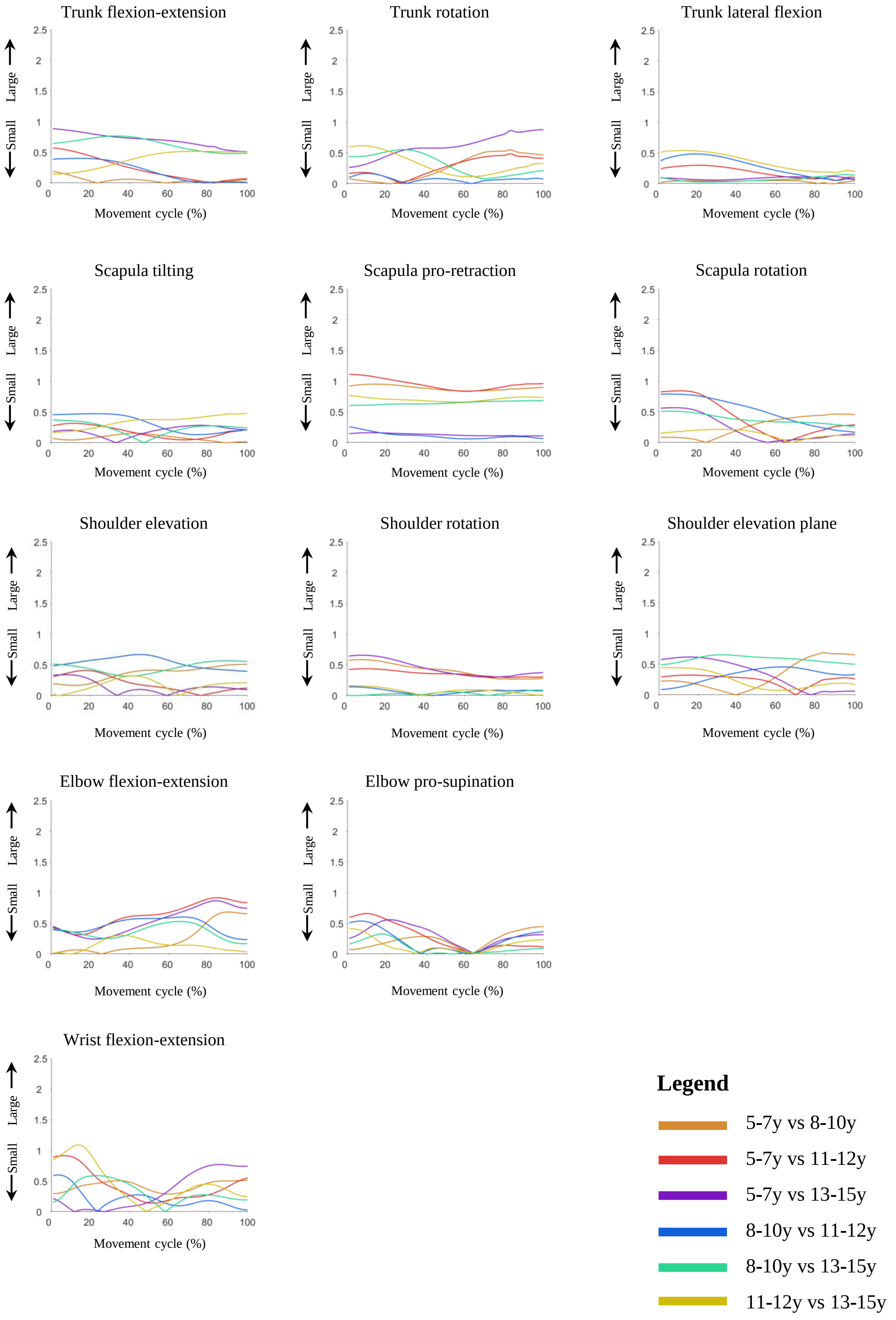

Fig S16. Hand to Shoulder (HTS)

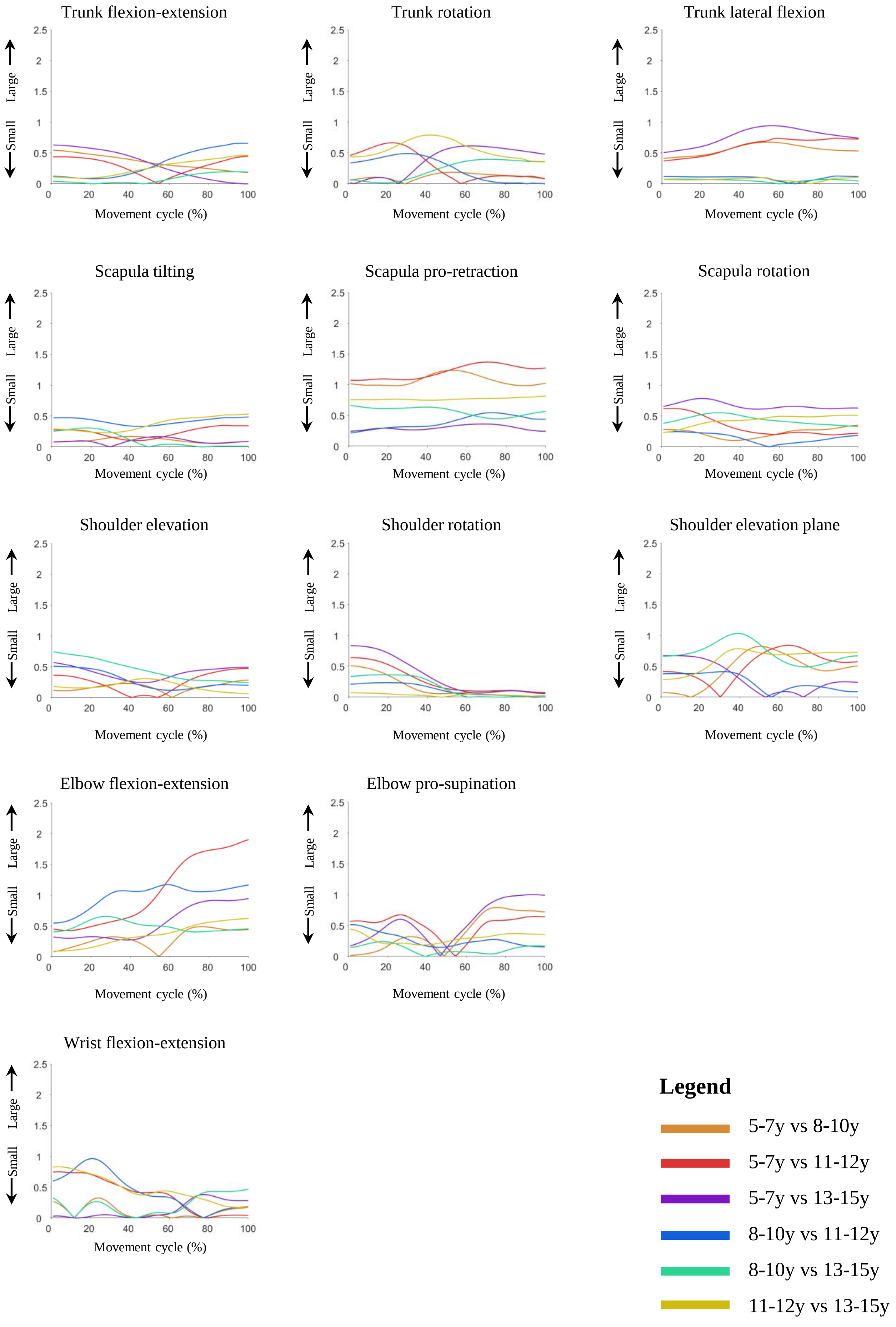

Supplement: S2 File — Joints are presented in rows, from top to bottom: trunk, scapula, shoulder, elbow, and wrist. Data is shown as effect sizes for every pair-wise post-hoc comparison after ANOVA test: 5-7y vs 8-10y (brown), 5-7y vs 11-12y (red), 5-7y vs 13-15y (purple), 8-10y vs 11-12y (blue), 8-10y vs 13-15y (green), and 11-12y vs 13-15y (yellow). Effect sizes are reported according to Cohen’s d criteria: small 0.2, medium 0.5, and large 0.8 over the movement cycle. (PDF) [file pone.0198524.s002.pdf]
